# Integrated multi-omics analysis and machine learning developed a prognostic model based on mitochondrial function in a large multicenter cohort for Gastric Cancer

**DOI:** 10.1186/s12967-024-05109-7

**Published:** 2024-04-23

**Authors:** Yimeng Ma, Jingjing Jin, Zixuan Xue, Jungang Zhao, Weiyang Cai, Wanli Zhang

**Affiliations:** 1 Department of Neurology, The First Affiliated Hospital of Wenzhou Medical University, Wenzhou, Zhejiang China; 2 Department of Clinical Laboratory, Key Laboratory of Clinical Laboratory Diagnosis and Translational Research of Zhejiang Province, The First Affiliated Hospital of Wenzhou Medical University, Wenzhou, Zhejiang China; 3 Department of Microbiology and Immunology, School of Basic Medical Sciences, Wenzhou Collaborative Innovation Center of Gastrointestinal Cancer in Basic Research and Precision Medicine, Wenzhou Key Laboratory of Cancer-Related Pathogens and Immunity, Wenzhou Medical University, Wenzhou, China; 4 Department of Urology, The Fourth Affiliated Hospital of Zhejiang University School of Medicine, Yiwu, 322000 China; 5 Department of Gastroenterology, The First Affiliated Hospital of Wenzhou Medical University, Wenzhou, Zhejiang China

**Keywords:** GC, Mitochondrial function, LETM2, mTOR pathway

## Abstract

**Background:**

Gastric cancer (GC) is a common and aggressive type of cancer worldwide. Despite recent advancements in its treatment, the prognosis for patients with GC remains poor. Understanding the mechanisms of cell death in GC, particularly those related to mitochondrial function, is crucial for its development and progression. However, more research is needed to investigate the significance of the interaction between mitochondrial function and GC cell death.

**Methods:**

We employed a robust computational framework to investigate the role of mitochondria-associated proteins in the progression of GC in a cohort of 1,199 GC patients. Ten machine learning algorithms were utilized and combined into 101 unique combinations. Ultimately, we developed a Mitochondrial-related-Score (MitoScore) using the machine learning model that exhibited the best performance. We observed the upregulation of LEMT2 and further explored its function in tumor progression. Mitochondrial functions were assessed by measuring mitochondrial ATP, mitochondrial membrane potential, and levels of lactate, pyruvate, and glucose.

**Results:**

MitoScore showed significant correlations with GC immune and metabolic functions. The higher MitoScore subgroup exhibited enriched metabolic pathways and higher immune activity. Overexpression of LETM2 (leucine zipper and EF-hand containing transmembrane protein 2) significantly enhanced tumor proliferation and metastasis. LETM2 plays a role in promoting GC cell proliferation by activating the mTOR pathway, maintaining mitochondrial homeostasis, and promoting glycolysis.

**Conclusion:**

The powerful machine learning framework highlights the significant potential of MitoScore in providing valuable insights and accurate assessments for individuals with GC. This study also enhances our understanding of LETM2 as an oncogene signature in GC. LETM2 may promote tumor progression by maintaining mitochondrial health and activating glycolysis, offering potential targets for diagnosis, treatment, and prognosis of GC.

**Supplementary Information:**

The online version contains supplementary material available at 10.1186/s12967-024-05109-7.

## Introduction

GC is the fifth most common cancer worldwide and the third leading cause of cancer-related deaths [1, 2] Particularly in China, GC is one of the most prevalent malignancies, with a high number of new cases and deaths reported [3]. Conventional treatments for GC, such as surgery and chemotherapy, are often ineffective due to frequent recurrence and metastasis, which results in poor prognosis [4, 5]. Metastasis, including peritoneal metastasis, lymph node metastasis, and distant metastasis, significantly contributes to the poor prognosis of GC patients [6]. Therefore, it is crucial to understand the molecular mechanisms driving GC progression.

Mitochondria, known as the “cellular power plants”, play a central role in cellular metabolism, including the tricarboxylic acid (TCA) cycle, fatty acid oxidation (FAO), electron transport chain (ETC), and oxidative phosphorylation (OXPHOS), which are involved in energy production and catabolism of biomolecules. Mitochondria also provide precursors for various biomolecules and adapt to different metabolic conditions by modulating key gene transcription [7]. Mitochondrial dysfunction is closely associated with various diseases, including cancer development and progression [8]. Therefore, investigating mitochondrial abnormalities and their roles in tumor cells may contribute to the development of novel and effective cancer therapies [9].

The role of mitochondria in the development of gastric cancer is increasingly emphasized. Studies have shown that mt-DNA mutations in gastric cancer may activate excessive DNA repair mechanisms, which not only alter the function of mt-DNA and its encoding genes but also that these mutations contribute to gastric cancer typing. This is clinically significant as it is expected to not only facilitate early screening and improved therapeutic management of patients but also aid in the discovery and application of precise molecular markers [10, 11]. Within the field of gastric cancer therapy, targeting strategies against mitochondria have shown significant clinical potential. For example, by targeting pyruvate dehydrogenase kinase 1 (PDK1), the PI3K/AKT/mTOR signaling pathway can be effectively inhibited, thereby inducing mitochondria-dependent apoptotic mechanisms and providing a new strategy for gastric cancer treatment [12]. Some researchers have also revealed how downregulation of NDUFS1, the largest subunit of ubiquinone oxidoreductase (complex 1) in the mitochondrial electron transport chain, promotes malignant progression of gastric cancer by activating the mROS-HIF1α-FBLN5 signaling pathway, emphasizing the critical role of targeting the mitochondrial components in gastric cancer therapy [13]. In summary, maintaining cellular and organismal homeostasis by regulating mitochondrial dynamics not only provides new perspectives for the prevention and treatment of gastric cancer but also the strategy of targeting mitochondrial dynamics is expected to become an effective approach for gastric cancer treatment as the research in this field progresses. Although this area of research is still in its preliminary stage, the establishment of a prognostic model for gastric cancer associated with mitochondrial changes is important for optimizing therapeutic regimens and improving patient survival. Therefore, further in-depth studies exploring the relationship between mitochondria and gastric cancer can not only reveal the pathogenesis of gastric cancer but also provide a theoretical foundation and experimental basis for the development of novel therapeutic strategies.

LETM2 is a recently discovered protein-coding gene predicted to be located in the inner mitochondrial membrane and predominantly expressed in the testis and spermatozoa. Both LETM1 and LETM2 belong to the SLC55 family of proteins, which act as mitochondrial cations and proton exchangers [14]. LETM1 is associated with poor prognosis in various malignant tumors [15, 16], whereas the biological role and mechanism of action of LETM2 in cancer remain poorly understood. LETM2 is located near the Wolfe-Congenital Megacolon Syndrome Candidate Gene-1 (WHSC1L1), which has been linked to Wolfe-Congenital Megacolon Syndrome, a rare inherited disorder characterized by intellectual disability, developmental delay, motor retardation, and epilepsy [17]. Zhou et al. demonstrated that the LETM2 regulated the PI3K-Akt signaling axis, which exhibited prognostic and therapeutic implications in pancreatic cancer [18]. However, there are no studies investigating the expression of LETM2 in GC and its functional roles. Hence, it is crucial to unravel the molecular mechanisms and develop a dependable classification model to evaluate prognosis and guide personalized treatment for individuals with gastric cancer (GC). This requires extensive research to understand GC progression and identify strong biomarkers that can accurately characterize patients and guide targeted therapies. By doing so, we can improve treatment outcomes and enhance overall patient care for GC. Through in-depth analysis of The Cancer Genome Atlas (TCGA) database, our study revealed that in gastric cancer tissues, the expression of LETM2 was significantly increased compared with that in normal tissues adjacent to the cancer. LETM2 expression was significantly increased in gastric cancer tissues compared to normal tissues adjacent to the cancer. Furthermore, we observed that in gastric cancer patients, the low-expression group of LETM2 showed a more optimistic prognosis compared to the high-expression group. This finding highlights the potential role of LETM2 in gastric cancer, not only as a key molecular marker for gastric cancer development but also as an important biomarker for predicting patient prognosis.

The mTOR signaling pathway plays a crucial role in cell growth, metabolism, and disease. Dysregulation of the mTOR pathway has been implicated in various diseases, including cancer, cardiovascular disease, and diabetes [19]. Several mTOR inhibitors, such as temsirolimus, everolimus, and lidamox, have been developed in clinical trials [20]. However, the limitations of efficacy and adverse effects restricted its clinical application. Therefore, there is an urgent need to develop combination or targeted therapies to expand the treatment options in the mTOR signaling pathway. The mTOR/Akt axis maintains energy balance through energy-generating activities, such as the Warburg effect, to meet the proliferative demands of GC cells. Glucose, as the main source of cellular energy, is taken up by cancer cells, leading to increased glycolytic flux and sustained growth and proliferation [21] to support the highly proliferative properties of GC cells.

Herein, we introduced a novel metric model MitoScore to forecast the efficacy and prognosis of therapeutic interventions in GC. Through our investigation, we discovered the heterogeneity among multi-cohort GC patients and evaluated their clinical outlook. Furthermore, LETM1 is an essential mechano-mediator that reprograms GC cell metabolism. We explored the clinical value and molecular mechanism of LETM2 in GC progression, which may serve as a potential therapeutic target in cancer management. We demonstrated that LETM2 promotes tumor progression by activating the mTOR pathway, thereby inducing mitochondrial homeostasis and facilitating glycolysis, ultimately leading to accelerated proliferation of GC cells.

## Materials and methods

### Data collection

The RNA-sequencing data and clinical information of GC patients were obtained from the TCGA (The Cancer Genome Atlas) database (https://portal.gdc.cancer.gov) and the GEO (Gene Expression Omnibus) dataset. A total of 1467 samples were included in the analysis, with 375 samples from TCGA-GC and 172 samples from the 1092 GEO dataset (GSE84437, GSE15459, GSE26899, GSE26901). Differentially expressed genes (DEG) were performed by using a limma package. Adjusted P-values (adjP) < 0.05 and fold change (FC) > 1 were considered to be significantly differentially expressed genes.

We focused on mitochondria-related genes by extracting a list of 1136 genes from the comprehensive mitochondrial gene database, MitoCarta 3.0 (https://www.broadinstitute.org/mitocarta/). Genes that were not present in the TCGA or GEO databases were excluded from our analysis. Please refer to Additional file 14: Table S1 for the specific gene list. We combined mRNA datasets and conducted a comprehensive screening process. We used the “Venn” tool to visually represent the overlap between DEGs associated with mitochondrial function and GC prognosis.

### Construction of MitoScore signature

To construct the MitoScore Signature, we used the NMF (Non-negative matrix factorization) algorithm to analyze the sectionalization of tumor samples based on the expression levels of 12 mitochondrial key genes via the NMF package. Furthermore, we integrated ten diverse machine learning algorithms and evaluated 101 algorithmic combinations [22, 23], including Support Vector Machine (SVM), Least Absolute Shrinkage and Selection Operator (Lasso), Gradient Boosting Machine (GBM), Random Forest, Elastic Net, Stepwise Cox, Ridge, CoxBoost, Super Partial Correlation (SuperPC), and Partial Least Squares with Cox regression (plsRcox). We developed a sequential approach that involved identifying the best prognostic variables using univariate Cox regression modeling. These algorithms were applied in the total TCGA GC cohort and verified in both the training and testing cohorts at the ratio of 3 to 7, and the best-performing model was constructed. This model was validated by both internal and external datasets.

Each patient’s MitoScore was calculated, and they were then divided into high- and low-subgroups based on the median score. The prognostic significance of the MitoScore signature was assessed using Kaplan–Meier (KM) survival analysis. To confirm its prognostic power, we further utilized it in the GSE84437, GSE62254, and GSE84426 datasets, which contained survival information of external GC datasets. Principal Component Analysis (PCA) and t-distributed Stochastic Neighbor Embedding (t-SNE) were used to analyze its predictive power.

### Construct a Predictive Nomogram.

To fully expand the predictive power of MitoScore-related signature, the nomogram was then constructed based on the clinical characteristics, including age, T, N, M, pathological stage, and MitoScore signature. Each patient could sum up the variable score and finally establish a predictor of survival. To validate the precision of the projected survival rates at 1-, 3-, and 5-year intervals, we generated calibration plots and ROC curves via ggDCA and timeROC package.

### ScRNA-seq data processing.

The GEO: GSE183904 dataset contains annotated cell types from each sample. We determined several cell types based on the annotation file.

Using UMAP, we identified different cell types, including T cells (CD4 + and CD8 +), B cells, cancer-associated fibroblasts, tumor-associated macrophages, tumor-associated endothelial cells, and epithelial cells. We analyzed the scRNA-seq data and determined metabolic pathway enrichment using the ReactomeGSA R package [38]. The gene expression levels for each cell type were quantified using the “AverageExpression” function in Seurat.

### Mutation landscape analysis.

The copy number variation (CNV) profiles of patients with GC patients were downloaded using the TCGA bio links package and divided into subgroups according to the cut-off line of the MitoScore model. GISTIC2.0 can identify regions of the genome that are significantly amplified or deleted in a set of samples, which facilitates the localization of the target somatic copy alteration in the model [24]. Additionally, we compared common somatic mutations in individuals with high- and low-MitoScore via dplyr package. The total number of non-synonymous somatic mutations per megabase within the whole genome was calculated to determine the TMB (tumor mutation burden).

### Biological function and pathway enrichment analysis.

In order to explore the biological functions and pathway processes associated with MitoScore, we conducted KEGG (Kyoto Encyclopedia of Genes and Genomes), GSVA (http://www.bioconductor.org), and GSEA (https://www.gsea-msigdb.org/gsea/msigdb/index.jsp) analysis using clusterProfiler, GSVA, and GSEABase packages. GSVA converts gene expression data from the level of an individual gene to the degree of enrichment of a gene set, by calculating the level of enrichment of the gene set in each sample.

### Assessment of immune microenvironment.

We used various bioinformatic algorithms, including ssGSEA [25], TIMER [26], CIBERSORT [27],CIBERSORT-ABS [28], QUANTISEQ [29], MCPcounter [30], Xcell [31] and EPIC [32], to comprehensively assess the immune infiltration level and molecular aspects. These algorithms estimated the abundance of different immune cell subpopulations using specific strategies. Additionally, we evaluated the tumor immune score using the ESTIMATE algorithm, which quantifies the immune activity or infiltration level based on gene expression profiles [33]. Furthermore, we analyzed the immune activity in different immune subtypes, such as wound healing (C1), IFN-g dominant (C2), inflammatory (C3), lymphocyte depleted (C4), immunologically quiet (C5), and TGF-b dominant (C6) [34]. We used the ssGSEA package to calculate different immune signatures by computing the enrichment or relative abundance of marker genes. We analyzed the expression patterns of 60 immunomodulatory genes, including those involved in antigen presentation, cell adhesion, co-inhibitors, co-stimulators, ligands, and receptors.

### Plasmids and Antibodies, Cells, laboratory animals

LETM2 overexpression plasmid and its control plasmid were purchased from Wuhan Miaoling Biotechnolog. The following antibodies were used to detect specific proteins: LETM2 LETM2 (1:1000) (proteintech 17180-1-AP), Santa Cruz Biotechnology provided antibodies against cyclin D1(1:1000) (sc-20044), CDK2 (1:2000) (sc-6248), CDK4(1:2000) (sc-260), CDK6(1:1000) (sc-7961). Cell Signaling Technology (CST) provided antibodies against LDHA(1:500) ( #3582), Glut1(1:1000) ( #73015), α-Tubulin(1:10000) ( #2144).AGS and SGC7901 are owned by our laboratory and were identified correctly by STR sequencing. The nude mice used in this study were all BALB/c strain mice, female, 3- 4 weeks old, purchased from Viton Lever Technology Co. and kept in the SPF grade. All animal studies were conducted according to the Association.

### ATP, Soft agar colony formation assay (Soft Agar)

First, transfer the cell seed plate to a 96-well plate. Add 25 μl of PBS to each well, followed by 25 μl of ATP substrate solution. Place the plate in a micro-oscillator and shake for 5 min. After shaking, transfer 40 μl of the mixture to a black 96-well plate. Keep the plate at room temperature, away from light, and let it sit undisturbed for 10 min. Next, turn on the computer and set up the parameters of the chemiluminescence instrument. Insert the black 96-well plate into the instrument and start the detection process. Once the test was completed, export the experimental data for analysis. Repeat the ATP experiment on days 1, 3, and 5 to gather data for different time points.

### Flow cytometry

Cells were first seeded into six-well plates, starved for 12 h, and collected after 24 h of full accompaniment, centrifuged at 1200 g/min for 2 min, discarded the supernatant, and the cells were resuspended by adding PBS and centrifuged at 1200 g/min for 2 min. Discard the supernatant, add 200 μl PI staining solution and 30 μl RNase per tube and mix well. The cell cycle was detected by flow cytometry.

Western blot assay Protein samples were loaded onto PAGE gels, concentrated at 150 V for 15 min, and separated at 120 V for 1.5 h. Proteins were transferred to membranes, which were subsequently blocked in 5% non-fat milk for 1 h and incubated in 1 × primary antibodies overnight at 4 °C. Membranes were washed in TBS and incubated in secondary antibody for 2.5–3 h at 4 °C, and were subsequently developed. Images were scanned, saved, and analyzed using a Typhoon 7000 film sweeping instrument.

### Statistical analyses

We performed all statistical analyses using the R Project software (https://www.r-project.org/, version 4.0.5). The Wilcoxon rank-sum test compared variables between two groups, the Kruskal–Wallis test of variance compared variables among multiple groups, and Spearman’s rank correlation analysis computed correlation coefficients. Fisher’s exact test or chi-square test compared contingency tables and categorical variables. Kaplan–Meier survival analysis with the log-rank test compared the prognosis between two subgroups, and the hazard ratio (HR) of variables was calculated using univariate and multivariate Cox proportional hazard regression analyses. The LASSO-Cox model was computed using the Glmnetr package, and the random forest survival algorithm was conducted using the randomForestSRC R package. Statistical significance was denoted as follows: *, p < 0.05; **, p < 0.01; ***, p < 0.0001; ns: not significant.

## Results

### Preliminary screening of MitoScore

**Fig. 1:**
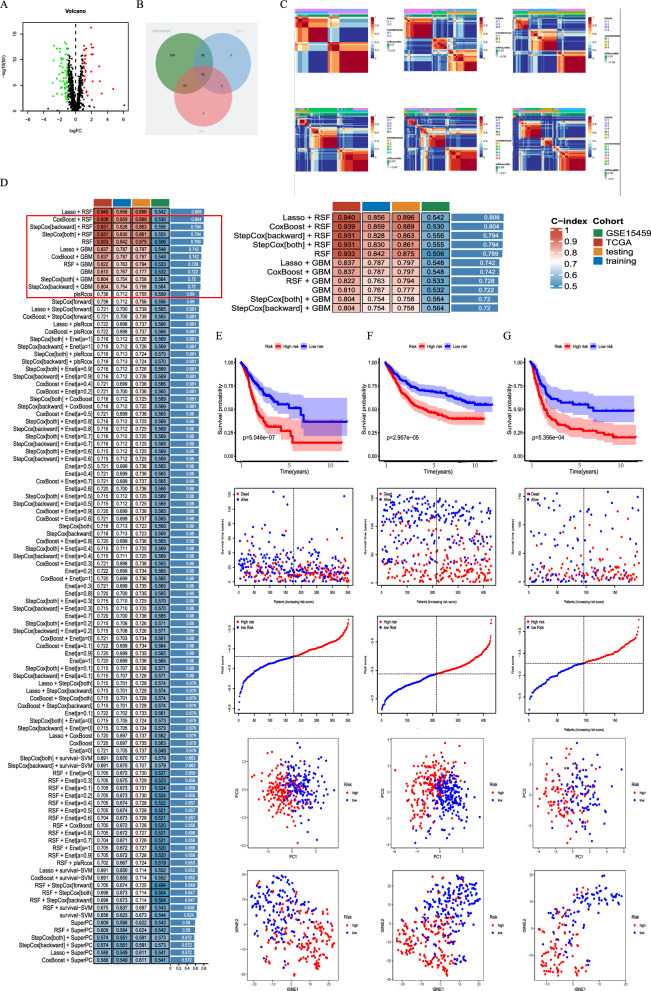
Preliminary screening of MitoScore signature. **A** Volcano plot of the differential expressed mitochondria-associated genes analysis in the GC and adjacent normal tissues. Red/blue dots represent upregulated/downregulated genes according to the criteria; **B** Venn plot of DEG, prognostic genes, and mitochondria-associated genes; **C** 101 different combinations of machine learning algorithms used by MitoScore. Each model’s c-index was calculated; Survival status, time distribution, tSNE analysis, and PCA analysis between two different subgroups

Expression data and clinical information were obtained from the TCGA and GEO databases in this study. We initially collected 1136 genes related to mitochondrial function from the MitoCarta3.0 database. Differential analysis was performed to identify genes with different expressions (DEGs) between normal and GC tissues (Fig. 1A). Then, a Venn plot was used to identify DEGs involved in both mitochondrial function and GC prognosis (Fig. 1B), resulting in the selection of 12 genes (ALDH3A2, ARMCX2, FKBP10, GCDH, GLS2, IDE, LETM2, OSBPL1A, POLRMT, QTRT1, SLC25A15, and TIMM8A). The expression patterns of these genes are shown in Additional file 1: Figure S1. Additionally, we analyzed to examine the expression levels and prognostic implications of these 12 genes across multiple cancer types. This pan-cancer analysis provides a comprehensive understanding of the gene expression patterns (Additional file 2: Figure S2A). And their potential impact on patient outcomes in various cancer types (Additional file 2: Figure S2B).

To develop more precise GC mitochondrial prognostic models, clustering analysis was performed on the activity values of the 12 signature genes. The most stable cluster, K = 2, was identified (Fig. 1C). Ten machine learning methods, including random survival forest, CoxBoost, support vector machine, gradient boosting machine, elastic net, LASSO-Cox, and their combinations, were employed. The average C-index of 101 algorithm combinations was evaluated in the total, training, and validation sets to identify the best models (Fig. 1D). Finally, the COX and RSF algorithms were used to build the MitoScore model (Fig. 1E). External validation datasets (GSE84437, GSE15459, GSE26899, GSE26901) were used to verify the optimal model. High-risk and low-risk groups were categorized based on the median scores of the respective cohorts for K-M survival analysis. The results showed that patients in the high-risk group had a significantly worse prognosis than those in the low-risk group (Fig. 1F, G and Additional file 1: Figure S3). The high MitoScore subgroup was associated with poor prognosis and good predictive performance of the model. PCA and t-SNE analyses revealed distinct dimensional variations between these two clusters. These findings highlight the excellent prognostic value and clinical significance of MitoScore (Fig. 1F, G and Additional file 3: Figure S3).

### Construction of a prognostic nomogram based on MitoScore

A prognostic nomogram based on MitoScore was constructed to assess the predictive usefulness of MitoScore in GC patients. Multivariate and univariate Cox regression analyses were performed to evaluate the predictive power of MitoScore. The nomogram integrated the MitoScore and important clinical features, providing a quantitative prediction of the prognosis for GC patients and aiding in clinical decision-making. To predict patient overall survival probabilities, the prognostic nomogram of TCGA GC patient, incorporated MitoScore, age, and TNM stage, was shown in Additional file 4: Figure S4A. The accuracy of the prediction model was verified using calibration curves, which demonstrated a good fit between the predicted and observed survival probabilities for 1-, 3-, and 5-year survival (Additional file 4: Figure S4B). The AUC values of the nomogram indicated a high diagnostic value for the model (Additional file 4: Figure S4C). Decision curve analysis (DCA) outcomes showed that the nomogram model yielded significant net benefits across a wide range of risks (Additional file 4: Figure S4D). Overall, these findings suggest that the nomogram model based on the MitoScore signature performs strongly in predicting the prognosis of GC patients.

### Single-cell sequencing of the MitoScore model

The siRNA sequencing dataset (GSE183904) from the GEO database was analyzed, which included 150,798 cells from GC patients and 150,798 cells from controls (NC). After filtration, a total of 25,576 cells were retained for further analysis. Among these cells, the first 4000 highly variable genes were selected for subsequent analysis.

To assign these genes to known cell lineages, marker genes identified in a previous study were used. Leveraging the UMAP algorithm, all cells were meticulously categorized into 10 immune clusters, providing a detailed classification (Additional file 5: Figure S5A). The expression patterns of the 12 key genes were visualized using t-SNE analysis (Additional file 5: Figure S5A). The comprehensive bubble plots depicted in Additional file 5: Figure S5 Billustrated the expression patterns of characterization marker genes associated with each of the 10 cell clusters. These plots provide insights into the expression levels of marker genes specific to each cell type, highlighting potential differences in expression patterns of these MitoScore genes.

### Annotation of clinical characteristics for the MitoScore

To further investigate the relationship between MitoScore and clinicopathological parameters, which validate the predictive ability of MitoScore on GC prognosis, we conducted a stratified analysis based on the expression of the 12 genes in relation to TNM stage, age, gender, tumor level, clinical molecular subtype, Her2 positivity, PI3CA mutation status, TP53 mutation status, and EGFR mutant status. As shown in the Additional file 6: Figure S6, we found no significant MitoScore differences among age, gender, and Her2 status. Surprisingly, we observed significant differences among many above classifications, including pathological type, PI3CA mutation status, EGFR mutant phenotype status, T-stage, N-stage, and M-stage between the high- and low-MitoScore subgroups.

Kaplan–Meier survival analyses were also performed to assess overall survival in different strata of clinical characteristics, including age (< 65 years or ≥ 65 years), gender, stage, and pathological subtype (Additional file 7: Figure S7). In each subgroup, the high-MitoScore subgroup had worse overall survival compared to the low-MitoScore subgroup. Notably, in patients with lymphatic metastases (N-stage), the difference in survival was significant, while in those without lymphatic metastases (N0), the survival difference was not significant. These results indicated that the MitoScore signature has robust predictive power for the multi-subgroup prognosis of GC.

### Analysis of potential biological mechanisms of MitoScore signature

**Fig. 2:**
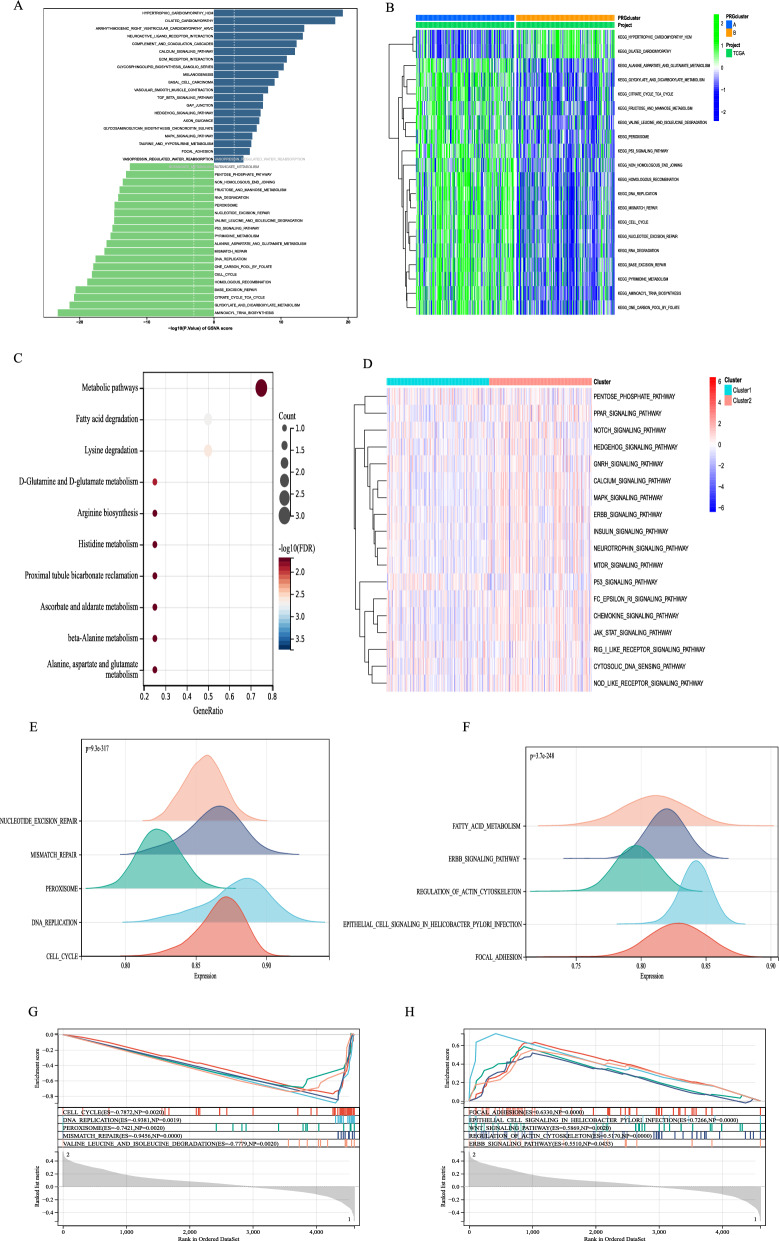
Functional enrichment analysis of MitoScore signature. **B** GSVA enriched pathways in the high- and low-MitoScore subgroup; **C** The KEGG enrichment analysis of the 15 key MitoScore signature genes; **D** GSEA enriched pathways in the high- and low-MitoScore subgroup; The top 5 GSEA enriched pathways in the** E** low- MitoScore subgroup and** F** high-MitoScore subgroup

To develop deeper into the biological processes linked to the MitoScore signature, we executed an enrichment analysis. The GSVA results indicated that MitoScore was primarily associated with many proliferation-related processes, such as DNA replication and cell cycle (Fig. 2A, B). They were also closely related to cellular metabolism, including glycometabolism and amino acid metabolism. Similar results were obtained using KEGG analysis. MitoScore signature appeared most highly concentrated in the metabolic relative pathways, such as fatty acid degradation, lysine degradation, and histidine metabolism (Fig. 2C). Furthermore, we utilized GSEA to identify potential pathways associated with MitoScore (Fig. 2D). The lower MitoScore subgroup showed significant enrichment for cellular process-related pathways, such as cell cycle, DNA replication, excision repair, and mismatch repair (Fig. 2E, G). In contrast, the higher-MitoScore subgroup was predominantly associated with cellular metabolic and signaling pathways, including fatty acid metabolism and ERBB signaling pathway (Fig. 2F, H). Based on these analyses, we found that our model was mainly enriched in cell metabolism, signaling pathway transduction, and cell proliferation. We offer a more comprehensive insight into the distinct biological pathways observed within the two subgroups of MitoScore in Additional file 8: Figure S8. Additionally, we provide a detailed analysis of the role played by each model gene in Additional file 9: Figure S9 across all KEGG pathways. This in-depth investigation allows for a thorough understanding of the specific biological mechanisms underlying the observed differences in MitoScore subgroups and the potential significance of each model gene in various KEGG pathways.

### Mutation landscape analysis of MitoScore signature

**Fig. 3:**
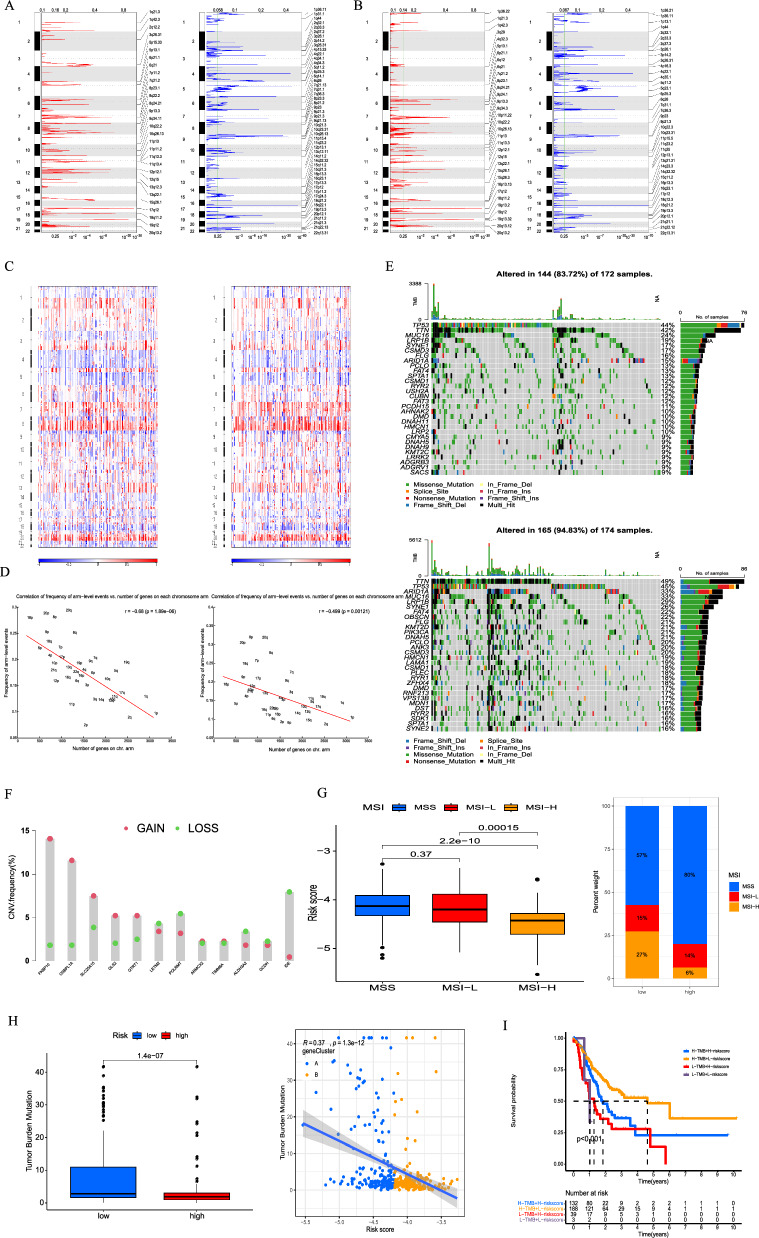
Mutation landscape analysis of MitoScore signature genes. Recurrent regions of copy number amplification and deletion in the **A** low and **B** high- MitoScore subgroup; **C** the Manhattan plot of copy number in high- (right) and low-(left) MitoScore subgroup; **D** correlation of frequency of arm-level events and number of genes on chromosome arm; **E** Top 15 mutated genes were illustrated in the low- (up)and high-(down) MitoScore signature; **F** the CNV frequency of each MitoScore signature genes; **G** The MitoScore of GC patients with microsatellite instability-high (MSI-H), microsatellite instability-low (MSI-L) and microsatellite stability (MSS); **H** Comparison of high- and low- MitoScore subgroups of TMB; **I** MitoScore and TMB-categorized OS Kaplan–Meier curves

To elucidate the genetic profiles of distinct MitoScore signatures, we investigated the distribution of somatic variants in patients with GC using the map tools package. We observed different mutation-driven patterns between the two MitoScore subgroups. Additionally, using GISTIC2.0, we identified the frequency of recurrent copy number alterations in the high- and low-MitoScore groups. We found that the higher MitoScore group displayed a higher frequency of recurrent copy number alterations compared to the lower subgroup. We also identified the most frequent and overexpressed amplification or deletion events at the Chr9 and Chr 11, respectively. (Fig. 3A, B). Notably, we observed a higher copy number in chr7 and chr8 in both the lower and higher MitoScore subgroups (Fig. 3C). There existed a close relation between the frequency of arm-level events and several genes on the chromosome arm (Fig. 3D).

We found that TTN, TP53, MUC16, and LRP1B have high mutation frequencies in both low-MitoScore and high-MitoScore (Fig. 3E). Meanwhile, copy number variation (CNV) plays an important role in the study of cancer occurrence and development. We found that the highest copy number variation (CNV) in this model was found in FKBP10 and OSBPL1A (Fig. 3F). Our study consistently emphasized the significance of the 12 MitoScore signature genes in GC. We presented a comprehensive evaluation of the diverse roles and characteristics of these genes in pan-cancer (Additional file 10: Figure S10).

GC is molecularly classified into four subtypes based on their molecular characteristics: EBV (EBV positive), MSI (microsatellite instability), GS (genomically stable), and CIN (chromosomal instability). Considering the significant role of TMB and MSI in determining individual response to immunotherapy, we examined their relationship with MtDEG. Our findings revealed that a high MSI in our model corresponded to a lower risk and a more favorable prognosis. Conversely, the microsatellite stable (MSS) status was higher in the high-risk group, and this model demonstrated statistical significance in relation to the MSI typology. (Fig. 3G). The results showed that MitoScore was markedly and negatively correlated with TMB, with patients in the low-MitoScore subgroup having higher TMB levels (Fig. 3H). TMB was significantly lower in the high-MitoScore subgroup than in the low- subgroup. The integration of two models, characterized by high TMB and low-risk MitoScore, resulted in a more favorable prognosis (Fig. 3I).

In summary, these results strongly suggested a close relationship between the MitoScore in GC. The combination of the MitoScore signature and TMB may serve as a valuable biomarker for predicting the prognosis of GC patients.

### MitoScore was associated with immune characterization and immunotherapy responses in GC

**Fig. 4:**
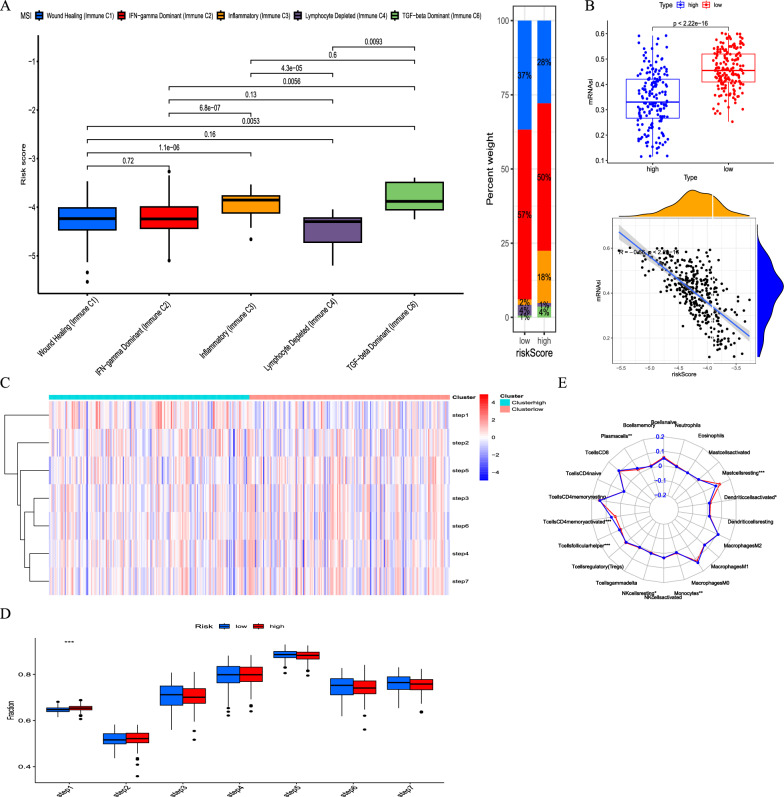
Correlation between the MitoScore and immune microenvironment. **A** Box plot portrays the dissimilarities in five cancer immunity subgroups between MitoScore subgroups; B. Expression pattern and correlation between mRNAsi and MitoScore signature; **C** Heatmap showed the expression levels of MitoScore signature in the seven-step Cancer-Immunity Cycle; **D** Box plot portrays the dissimilarities in the seven-step Cancer-Immunity Cycle between two MitoScore subgroups; **E** The differences in immune cell subpopulations between the high and low MitoScore subgroup

The tumor immune microenvironment plays a crucial role in the therapeutic effects and prognosis of patients with malignant tumors. Using ssGSEA cluster analysis, we classified GC samples into five subgroups: C1 (wound healing), C2 (IFN-gamma dominant), C3 (inflammation), C4 (lymphocyte depletion), and C6 (TGF-beta dominant). Simultaneously, we conducted an analysis of the immunophenotyping of each gene (Additional file 11: Figure S11). KM curves demonstrated significant prognostic differences between these subtypes (Fig. 4A). Specifically, significant differences were observed in the C1 (wound healing) and C3 (inflammation) subgroups. Cancer stem cells (CSCs) are a subpopulation of cancer cells that exhibit characteristics associated with normal stem cells and can give rise to different cell types within a tumor. To investigate the gene expression and epigenetic characteristics of CSCs, we calculated the mRNAsi scores of each GC sample. There was a strong correlation and variability between mRNAsi scores and MitoScore. Specifically, higher MitoScore values were associated with lower mRNAsi scores (Fig. 4B).

Seven phases of the tumor immune process are additionally involved phase 1 (antigen release), phase 2 (cancer antigen presentation), phase 3 (priming and activation), phase 4 (tumor immunized infiltrating cells recruitment), phase 5 (immune tissues influx), phase 6 (cancer cells comprehension by T cells), and phase 7 (cancer cells executing).

Additionally, we found that there was a stronger involvement of the tumor immune process in the first phase in high MitoScore samples (Fig. 4C, D).

**Fig. 5:**
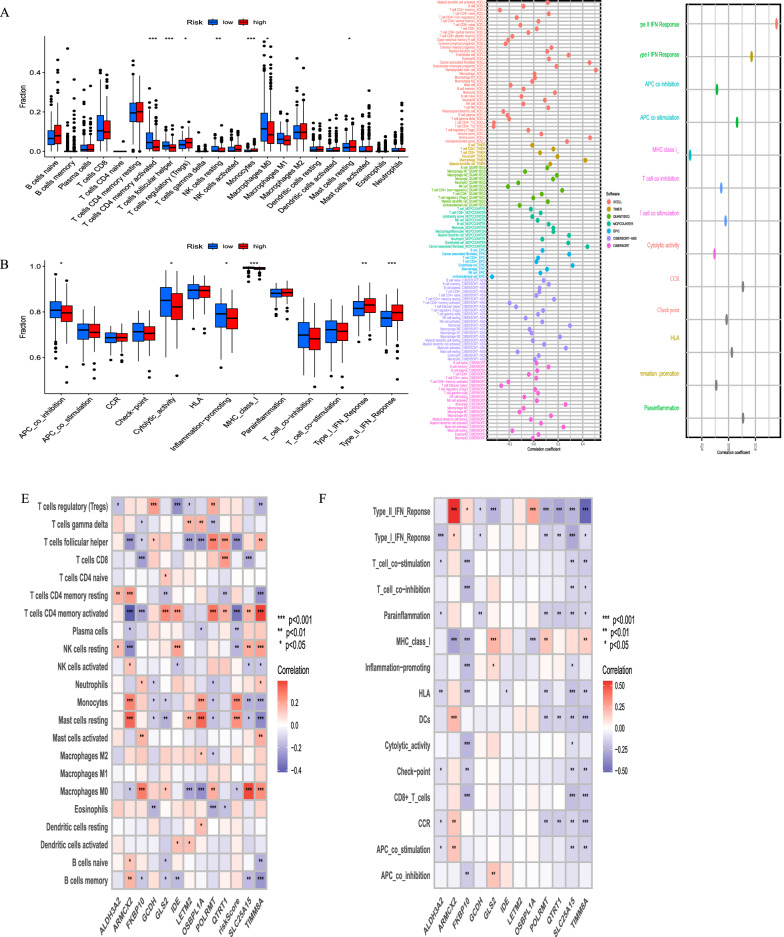
Correlation between the MitoScore and immune infiltration cells. **A** infiltrating immune cells and **B** immune-related functions in the high and low MitoScore subgroups; Correlation analysis to estimate the presence of infiltrating immune cells **C** and immune-related functions **D**; The heatmap presented the summary of the correlation between MitoScore signature genes expression **E**, immune-related functions **F** and 22 immune cell types infiltrates

Considering the essential role of immune infiltration in tumorigenesis, we assessed the differences in immune cell subpopulations between the higher and lower MitoScore subgroups (Fig. 4E, Fig. 5A, C). Specifically, the higher MitoScore subgroup had a lower proportion of T cells CD4 memory activated and T cells follicular helper. Conversely, the lower MitoScore subgroup had a lower proportion of NK cells resting, monocytes, and mast cells resting. Furthermore, we explored the differences in the expression of immunological function between the high and low MitoScore subgroups. Our analysis revealed that the high-MitoScore subgroup exhibited greater immunological function. We observed higher levels of Type I IFN Response, Type II IFN Response, and Mast cells in the high MitoScore group. Conversely, the low MitoScore group exhibited higher levels of APC co-inhibition and Cytolytic activity (Fig. 5B, D). At the same time, we also explored the relation of MitoScore and immunomodulators, such as antigen-presenting, cell-adhesion, co-inhibitors, co-stimulatory factors, ligands, and receptors (Additional file 12: Figure S12A). We also conducted a detailed examination of the distribution of the 12 hub genes in terms of immune cells, immune functions, and immune modulators, providing valuable insights for future studies (Fig. 5E, F, Additional file 12: Figure S12B).

### LETM2 promoted the proliferation ability of GC cells

**Fig. 6:**
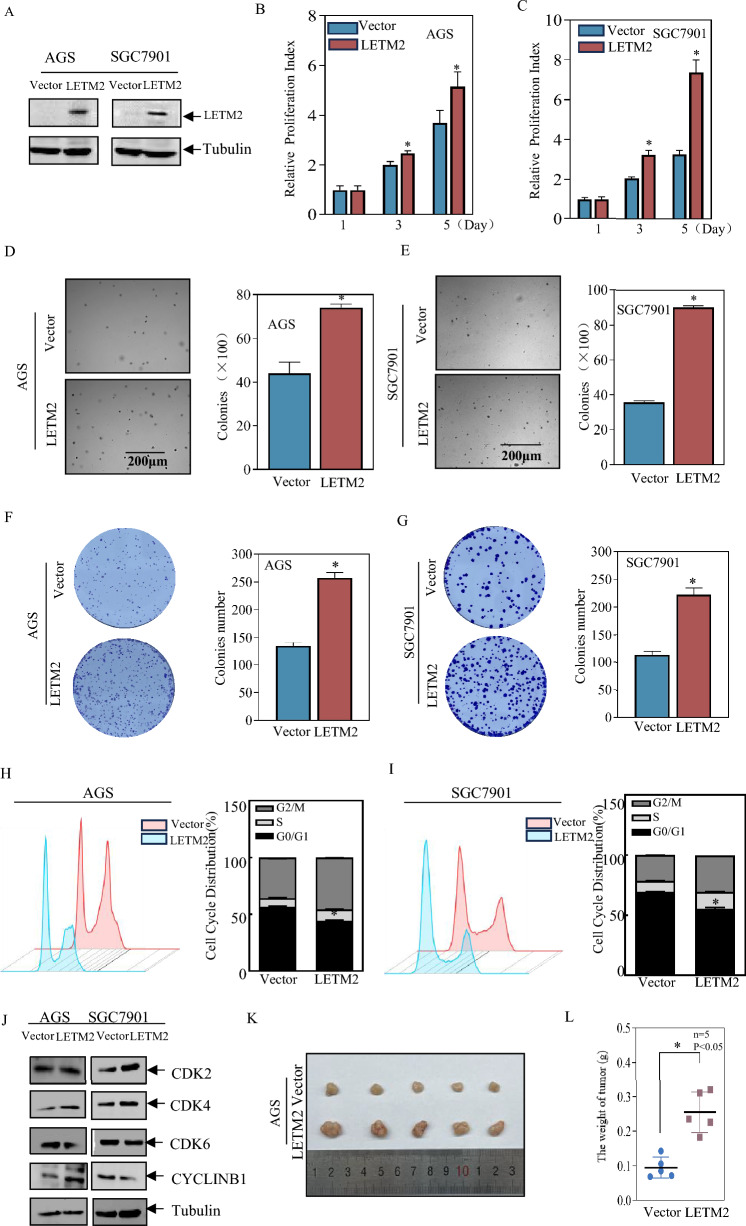
LETM2 significantly promoted the proliferation and metastatic ability of gastric cancer cells. **A** Identification of stably transfected cells overexpressing exogenous LETM2. **B** ATP proliferation rate assay to detect the effect on AGS cell proliferation after overexpression of LETM2. **C** ATP proliferation rate assay to detect the effect of overexpression of LETM2 on the proliferation of SGC7901 cells. **D**, **E** soft agar assay to detect the effect on the anchorage-independent growth capacity of AGS、 SGC7901 cells after overexpression of LETM2. **F**, **G** Plate cloning assay to detect the proliferation of ability of AGS、SGC7901 cells after overexpression of LETM2. **H** Flow cytometry detection of AGS(Vector), AGS(LETM2) cell cycle distribution. **I** Flow cytometry detection of SGC7901(Vector), SGC7901(LETM2) cell cycle distribution. **J** Western Blot experiments were performed to detect the relevant cyclins that regulate the G0/G1 phase. **K** Subcutaneous transplanted tumors were photographed. **L** Tumors were weighed

To investigate the potential role of LETM2 in GC development, we successfully created LETM2-overexpressing stable cell lines in AGS and SGC7901 cells. The overexpression efficiency of exogenous LETM2 was confirmed through Western Blot assay (Fig. 6A). We then assessed the proliferation rate of AGS and SGC7901 cells overexpressing LETM2 using the ATP proliferation rate assay on days 1, 3, and 5. The results demonstrated that LETM2 significantly promoted the proliferation ability of GC cells in vitro (Fig. 6B, C). Additionally, soft agar and plate cloning assays revealed that LETM2 promoted the anchorage-independent growth ability of GC cells (Fig. 6D, G).

Furthermore, we examined the cell cycle distribution of LETM2-overexpressing cells and control cells using flow cytometry. We observed a significant acceleration of the G0/G1 phase in the experimental group cells overexpressing LETM2 (Fig. 6H, I). Considering the impact of LETM2 on cell cycle progression in gastric cancer, we conducted Western Blot experiments to evaluate the expression of G0/G1 phase-associated cyclins, CDK2, CDK4, CDK6, CyclinB1, and CyclinE2. The results showed upregulation of CDK4 (Fig. 6J).

To better understand the effect of LETM2 on the proliferation ability of GC cells in a complex in vivo environment, we established a nude mice subcutaneous xenograft tumor model. Compared to control cells, the experimental group cells overexpressing LETM2 exhibited significantly higher tumor weight (Fig. 6K, L). The proliferative ability of gastric cancer cells was inhibited after knocking down the cell lines simultaneously (Additional file 13: Figure S13).These in vivo findings further support the inhibitory role of LETM2 in GC cell proliferation.

### LETM2 exerted a tumor-promoting effect by increasing mitochondrial ATP and glycolysis

**Fig. 7:**
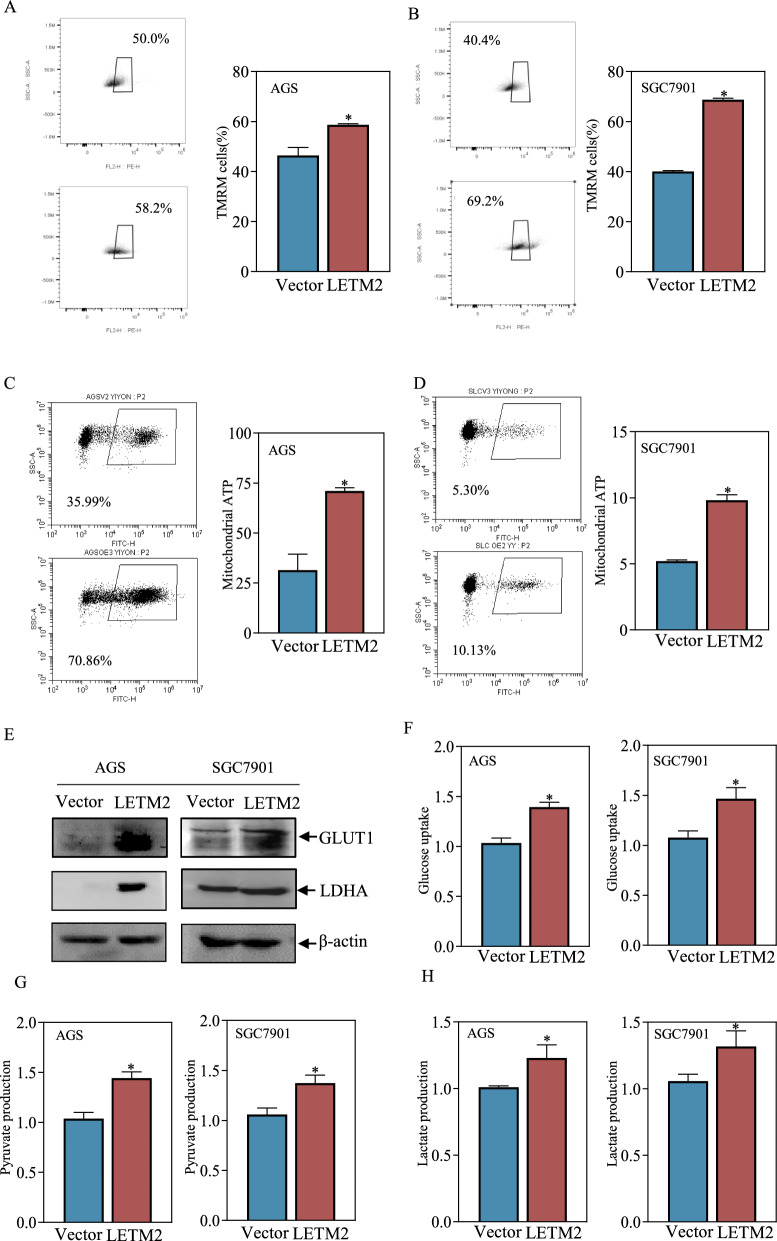
LETM2 exerts pro-carcinogenic effects by enhancing mitochondrial and glycolytic functions. **A** Flow cytometry detection of mitochondrial membrane potential in AGS(Vector), AGS(LETM2). **B** Flow cytometry detection of SGC7901(Vector), SGC7901(LETM2) mitochondrial membrane potential. **C** Flow cytometry detection of mitochondrial ATP in AGS(Vector), AGS(LETM2). **D** Flow cytometry detection of mitochondrial ATP in SGC7901(Vector), SGC7901(LETM2). **E** Western Blot assay detected related proteins regulating glycolysis **F** Enzyme labeling assay for AGS(Vector), AGS(LETM2) glucose uptake levels. **G** ELISA for SGC7901(Vector), SGC7901(LETM2) glucose uptake. **H** ELISA for AGS(Vector), AGS(LETM2) Pyruvate, Lactate Production **I** ELISA for SGC7901(Vector), SGC7901(LETM2) Pyruvate, Lactate Production

Given that LETM2 is a nuclear-encoded mitochondrial inner membrane protein, we hypothesized that LETM2 may impact mitochondrial function, thereby affecting the proliferation of GC cells. To investigate this, we examined the changes in mitochondrial membrane potential and mitochondrial ATP levels in GC cells overexpressing LETM2 using flow cytometry. The results revealed a significant increase in both mitochondrial membrane potential and mitochondrial ATP in LETM2-overexpressing cells (Fig. 7A, D).

Mitochondrial respiration plays a crucial role in providing substrates for glycolysis. We theorized that glycolysis might be upregulated to compensate for the increased mitochondrial function, consequently promoting the proliferation of GC cells. To validate this hypothesis, we assessed the impact of LETM2 overexpression on glycolysis in GC cells by analyzing the levels of the expression of key glycolysis-related proteins, glucose transporter 1 (GLUT1), and lactate dehydrogenase A (LDHA), using Western blotting. The results showed that LETM2 overexpression increased the protein expression levels of GLUT1 and LDHA (Fig. 7E). We also tested the ability of glucose uptake, pyruvate production, and lactate production. We found the levels of glucose uptake, pyruvate production, and lactate production were significantly higher in LETM2-overexpressing cells compared to the control group, indicating an enhanced glycolytic activity (Fig. 7F–I).

In summary, these findings suggested that LETM2 overexpression elevates mitochondrial function, leading to an increase in glycolysis and promoting the proliferation of GC cells.

### LETM2 promoted GC cell proliferation by activating mitochondrial transcription factors and glycolysis via the mTOR pathway.

**Fig. 8:**
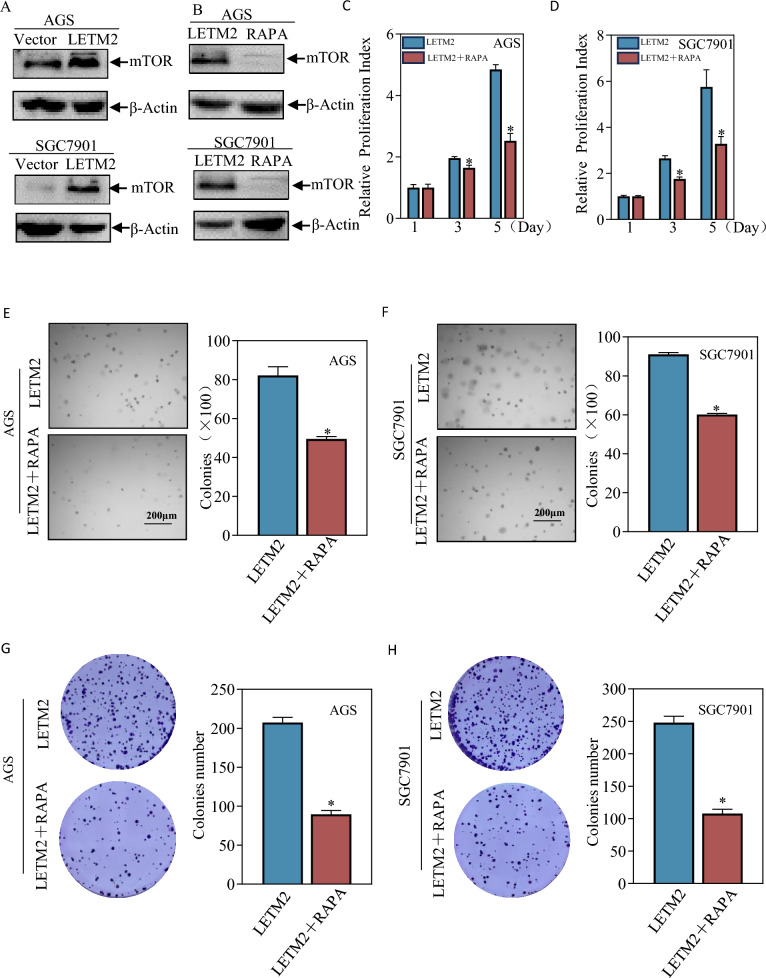
LETM2 exerts pro-carcinogenic effects by activating the MTOR pathway. **A** Western Blot assay to detect changes in the MTOR pathway after overexpression of LETM2. **B** Western Blot assay to detect changes in the MTOR pathway after treatment with rapamycin. **C** ATP proliferation rate assay to detect the effect of treatment with rapamycin on AGS cell proliferation. **D** ATP proliferation rate assay to detect the effect of rapamycin treatment on the proliferation of SGC7901 cells. **E** soft agar assay to detect the effect of treatment with rapamycin on the anchorage-independent growth capacity of AGS cells. **F** soft agar assay to detect the effect of treatment with rapamycin on the anchorage-independent growth capacity of SGC7901 cells **G** plate cloning assay to detect the effect of treatment with rapamycin on the proliferation of AGS cells. **H** Plate cloning assay to detect the effect of treatment with rapamycin on the proliferation of SGC7901 cells

Considering that mTOR is crucial for maintaining mitochondrial oxidative function and plays a role in glucose metabolism, we investigated whether LETM2 overexpression activates the mTOR pathway. We demonstrated the activation of the mTOR pathway upon LETM2 overexpression (Fig. 8A). To confirm that mTOR pathway activation is responsible for maintaining mitochondrial function and promoting glycolysis, we employed rapamycin to inhibit the mTOR pathway (Fig. 8B). Interestingly, we observed a deceleration in tumor cell growth upon treatment with rapamycin. This inhibitory effect was evident through the ATP proliferation rate detection assay, soft agar assay, and plate cloning assay, where rapamycin significantly suppressed the proliferative ability of GC cells (Fig. 8C–H).

These findings suggest that LETM2 overexpression activates the mTOR pathway, which in turn contributes to the maintenance of mitochondrial function and facilitates glycolysis. Furthermore, the inhibition of the mTOR pathway through rapamycin treatment leads to a notable reduction in the proliferation of GC cells.

## Discussion

GC is a complex and heterogeneous disease, making it challenging to predict patient prognosis. Prognostic factors are crucial in estimating disease progression, selecting appropriate treatments, and determining overall survival rates. Machine learning techniques are increasingly being used to predict cancer patient survival; however, effectively implementing them in clinical practice while maintaining accuracy is still a challenge. In this study, we utilized a novel computational framework and collected expression files from 1176 GC patients across five multi-cohorts worldwide to explore the correlation between mitochondrial function and prognosis in GC. Our model identified 12 genes (ALDH3A2, ARMCX2, FKBP10, GCDH, GLS2, IDE, LETM2, OSBPL1A, POLRMT, QTRT1, SLC25A15, TIMM8A), referred to as mitochondrial differentially expressed genes, which play a significant role in prognosis. We found that MitoScore can be a valuable tool for guiding therapeutic decisions and improving patient prognosis. By identifying these genetic alterations through MitoScore, clinicians can gain important insights into potential molecular mechanisms.

Mitochondrial dysfunction is a hallmark of cancer and a risk factor for gastric carcinogenesis. Therefore, identifying effective mitochondria-associated biomarkers for predicting the prognosis of GC patients is an encouraging research direction. Mitochondria are crucial energy suppliers of eukaryotic cells, acting as cellular stress sensors that regulate cell signaling, metabolism, and other biological processes through the production of reactive oxygen species (ROS) and the modulation of metabolites. Mitochondrial dysfunction is strongly associated with various diseases, including cancer development and progression [35]. Specific mitochondrial aberrations, such as oxidative damage, impaired ATP synthesis, disturbed calcium metabolism, mitochondrial DNA damage, and mitochondrial outer membrane permeability disorders, are unique to tumor cells and may increase programmed cell death or apoptosis [36, 37]. Targeting mitochondria has emerged as a promising approach for cancer therapy, with various inhibitors being developed and tested in clinical trials [38–40].

LETM2, a mitochondria-associated protein, has been found to be highly expressed in GC tissues, but its role in GC progression remains unclear. In our experiments, we discovered that overexpression of LETM2 promoted GC cell proliferation and metastasis both in vitro and in vivo. We hypothesized that LETM2 affects GC cell proliferation by affecting mitochondrial function. Supporting this hypothesis, we observed an increase in mitochondrial membrane potential, and mitochondrial ATP levels, upon LETM2 overexpression, suggesting improved mitochondrial health. Mitochondrial respiration provides the necessary substrates for glycolysis, leading us to theorize that compensatory glycolysis may be upregulated to promote the proliferation of GC cells. Indeed, we found enhanced glycolytic capacity upon LETM2 overexpression. Thus, we propose that LETM2 promotes GC cell proliferation by increasing mitochondrial homeostasis and enhancing glycolysis. Further analysis revealed that LETM2 is enriched in the mTOR pathway, a nutrient sensor that regulates mitochondrial oxidative function. mTOR is essential for maintaining mitochondrial respiratory capacity and plays a role in controlling cancer cell metabolism by regulating anabolic processes such as ribosome biogenesis and protein, nucleotide, fatty acid, and lipid synthesis [19, 41–43].

Glucose is the primary source of cellular energy, and cancer cells increase glucose uptake and glycolytic flux to sustain growth and proliferation. We hypothesized that LETM2 may activate the mTOR pathway to improve mitochondrial health and enhance glycolysis. To test this hypothesis, we treated GC cells overexpressing LETM2 with the mTOR inhibitor Rapamycin and observed a reduction in the proliferative capacity of these cells.

In summary, starting from the central molecule of energy regulation, mTOR, we discovered that LETM2 overexpression promotes mitochondrial respiratory capacity, glycolytic function, and GC proliferation through mTOR activation. mTOR signaling is commonly activated in tumors and controls cancer cell metabolism by altering expression or activity [17]. By utilizing mTOR inhibitors in patients with high LETM2 expression, we can provide a theoretical basis for their use in the treatment of GC patients.

## Conclusion

In summary, our study has successfully developed a MitoScore signature, which was subsequently validated in five external cohorts, demonstrating its superior predictive performance. Importantly, the MitoScore remained a powerful prognostic indicator even after adjusting for potential confounding factors, outperforming established clinical models. Furthermore, our investigation into the association between MitoScore and tumor immune microenvironment provides valuable insights for future in-depth studies. Moreover, our research identified LETM2 as a risk factor for GC prognosis, suggesting its potential as a promising prognostic indicator.

### Supplementary Information

**Additional file 1: Figure S1.** The expression pattern of **MitoScore** signature genes in GC.

**Additional file 2: Figure S2.** The expression pattern and survival analysis of MitoScore signature genes in pan-cancer analysis. A. Summarizes the expression difference between normal and cancer groups; B. Summarizes the survival difference between high- and low-gene expression groups.

**Additional file 3: Figure S3.** GSE26899 (A) and GSE26901 (B) survival curve. Survival status, time distribution, tSNE analysis, and PCA analysis between two different subgroups.

**Additional file 4: Figure S4.** Building nomogram: A. Nomogram to predict 1-, 3-, and 5-year GC patient survival; B.Nomogram calibration curves for 1-, 3-, and 5-year OS; C. AUC analysis of each variable included in the nomogram model; D. DCA curves were compared over a period of 1 year, 3 years, and 5 years for patients with GC.

**Additional file 5: Figure S5.** Uniform manifold approximation and projection (UMAP) plot of each **MitoScore** signature gene, cell types from gastric tumors cells from GEO: GSE183904.

**Additional file 6: Figure S6.** The expression of MitoScore signature in different clinical subgroups.

**Additional file 7: Figure S7.** Kaplan-Meier survival analyses of MitoScore signature in different strata of clinical characteristics.

**Additional file 8: Figure S8.** KEGG pathway activity between high- and low-MitoScore subgroups based on ssGSEA algorithm.

**Additional file 9: Figure S9.** The heatmap of KEGG pathway activity of each MitoScore signature genes.

**Additional file 10: Figure S10.** The landscape of MitoScore signature genes in pan-cancer analysis. A. Correlations between methylation and mRNA expression of inputted genes in the pan cancers; B. the methylation difference between tumor and normal samples in the pan cancers; C. correlations between CNV and mRNA expression in the pan cancers; D. copilot of the signature gene mutation frequency in pan cancers; E. copilot of the single-nucleotide variant in pan cancers.

**Additional file 11: Figure S11.** Box plot portrays the dissimilarities in the cancer immunity subgroup between MitoScore signature genes.

**Additional file 12: Figure S12.** Correlation between the MitoScore and immune infiltration molecular. A. The correlation of immune molecular and MitoScore; B. Correlation analysis to estimate the presence of infiltrating immune molecular and MitoScore signature genes.

**Additional file 13: Figure S13.** Knockdown of LETM2 inhibits the proliferative capacity of gastric cancer cells in vitro. (A) Knockdown LETM2 cell lines were identified. (B) ATP proliferation rate assay to detect the effect on AGS cell proliferation after knocking down LETM2. (B) ATP proliferation rate assay to detect the effect on AGS cell proliferation after knockdown of SGC7901. (D) Plate cloning assay to detect the effect on the proliferative ability of AGS and SGC7901 cells after knocking down LETM2. (E) soft agar assay to detect the effect on the anchorage-independent growth ability of AGS, SGC7901 cells after overexpression of LETM2.

**Additional file 14.** All of the included Mitochondria-related genes.

## Data Availability

The datasets used and/or analyzed during the current study are available from the TCGA (https://www.cancer.gov/aboutnci) and GEO (https://www.ncbi.nlm.nih.gov) databases.
