# Supplementary figures and images for: Integrated multi-omics analysis and machine learning developed a prognostic model based on mitochondrial function in a large multicenter cohort for Gastric Cancer
Source: J Transl Med. 2024 Apr 23;22:381. doi: 10.1186/s12967-024-05109-7 (PMC11040813; doi:10.1186/s12967-024-05109-7)

A

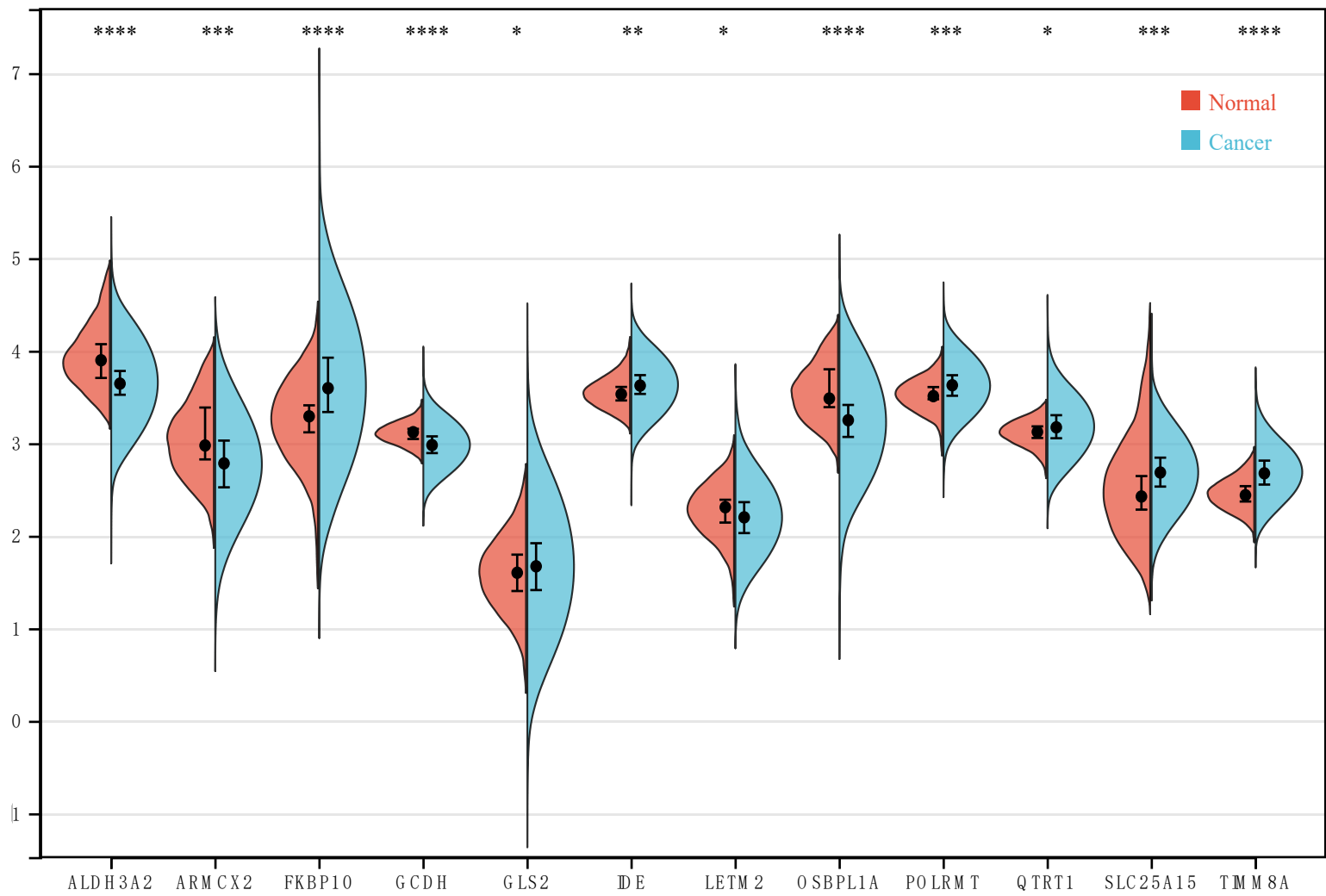

B

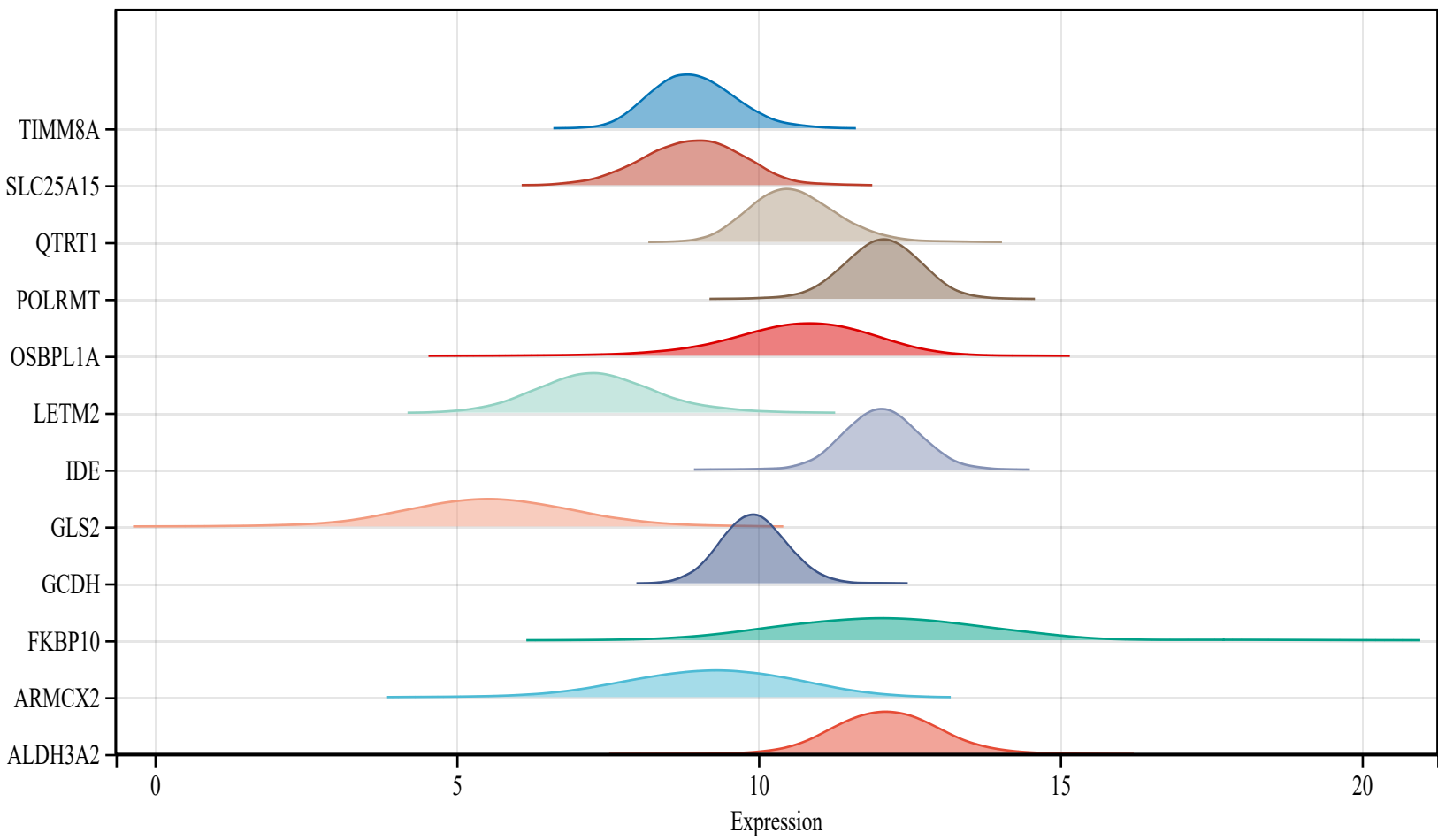

Supplement: Supplementary file 1 — Additional file 1: Figure S1. The expression pattern of MitoScore signature genes in GC. [file 12967_2024_5109_MOESM1_ESM.pdf]

A

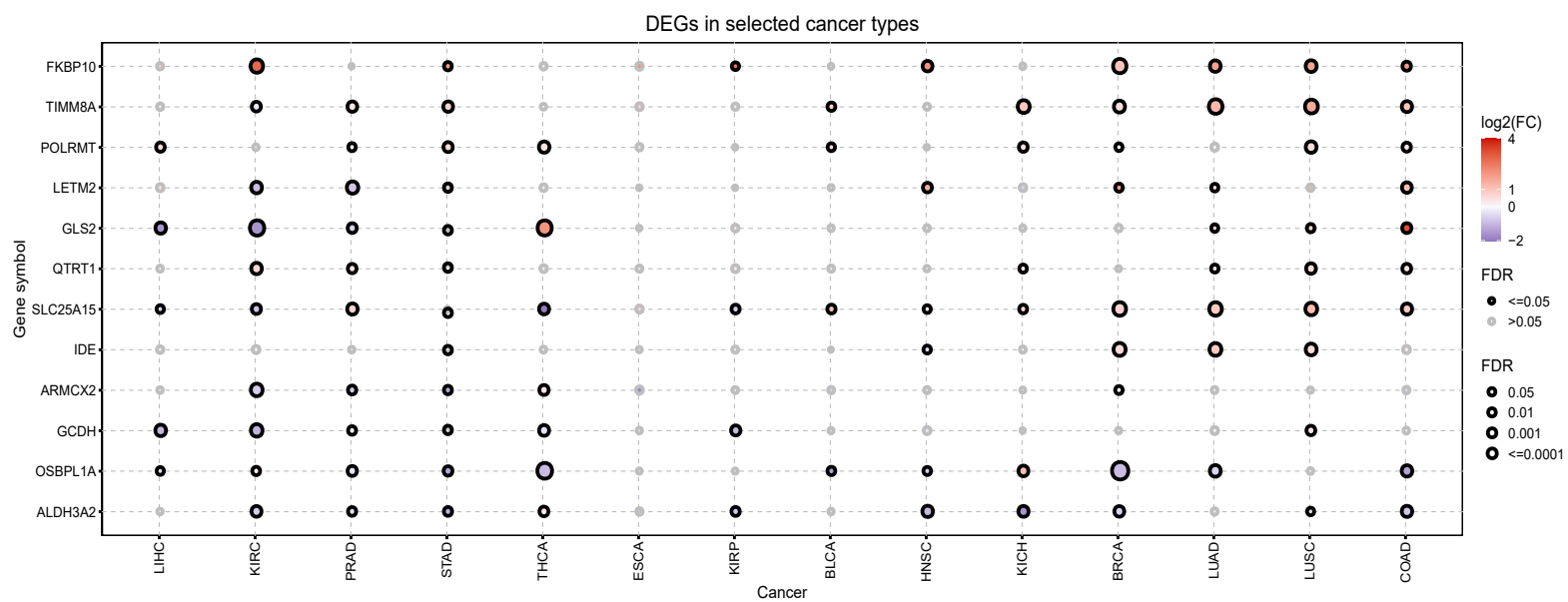

B

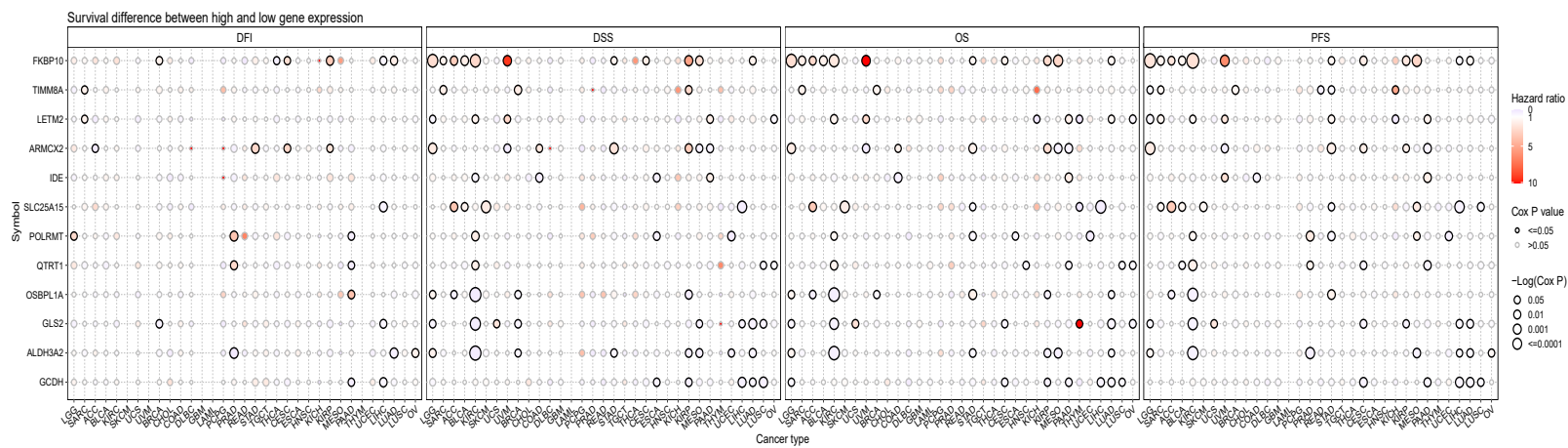

Supplement: Supplementary file 2 — Additional file 2: Figure S2. The expression pattern and survival analysis of MitoScore signature genes in pan-cancer analysis. A. Summarizes the expression difference between normal and cancer groups; B. Summarizes the survival difference between high- and low-gene expression groups. [file 12967_2024_5109_MOESM2_ESM.pdf]

A

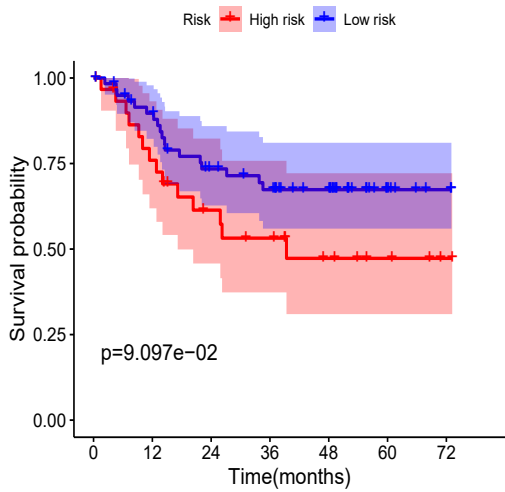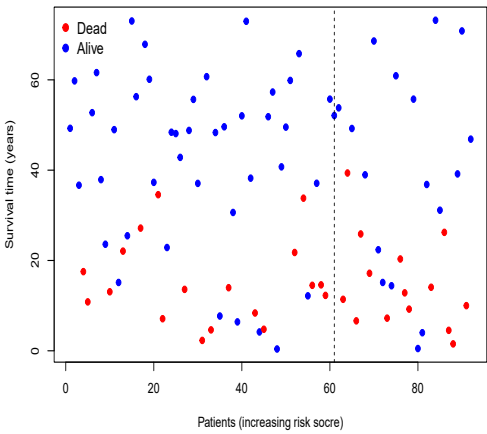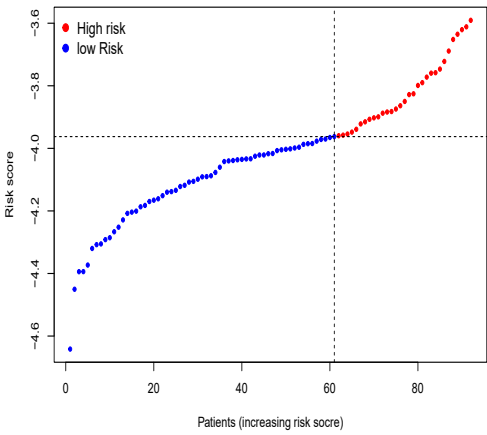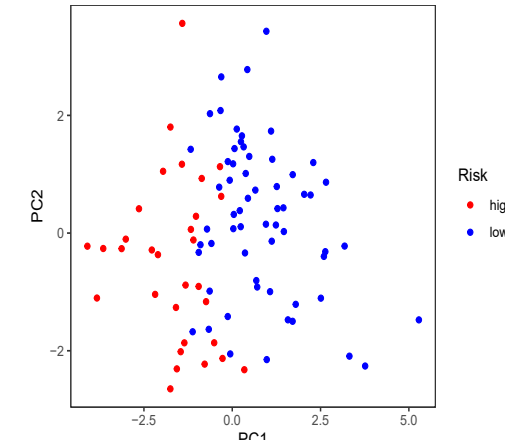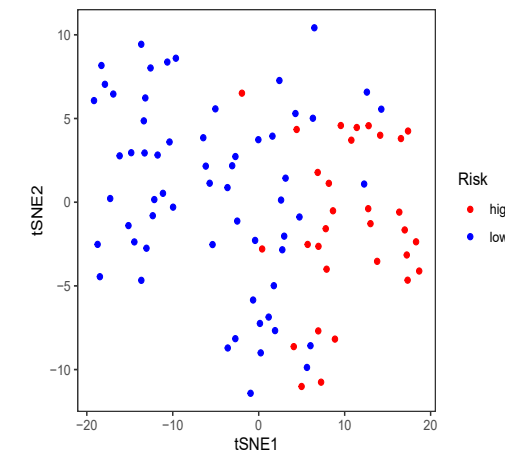

B

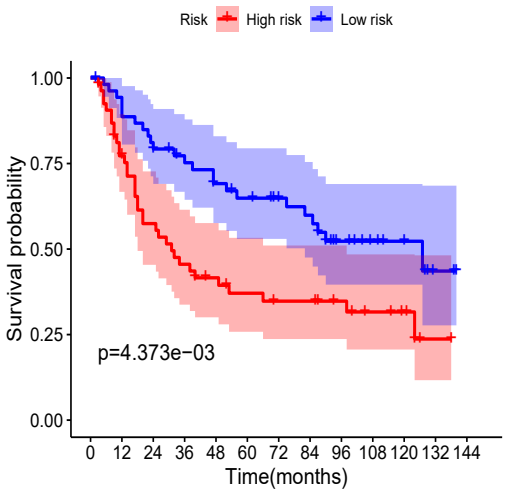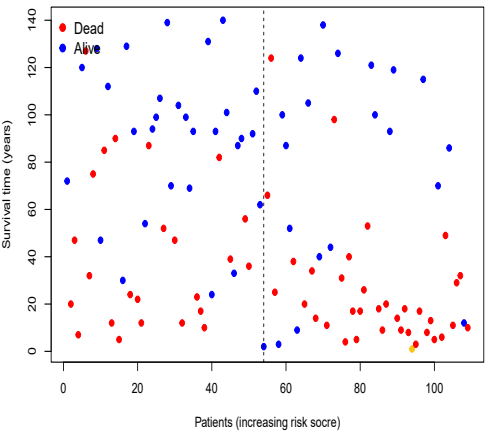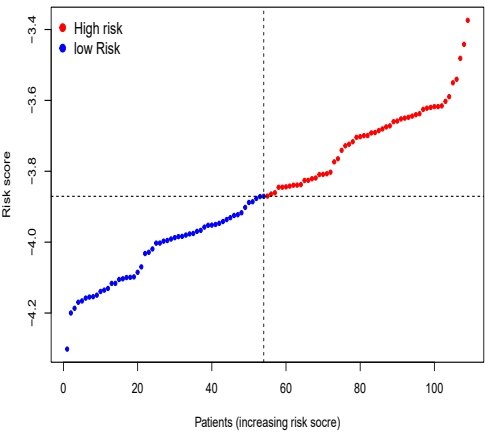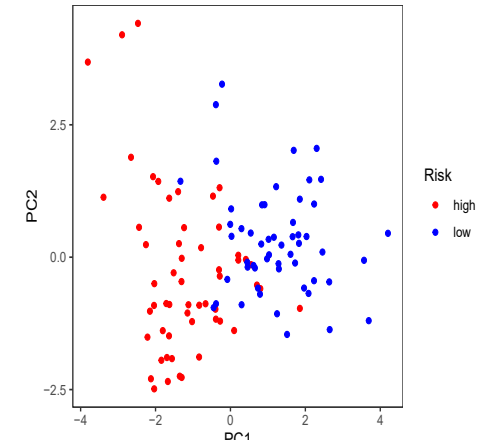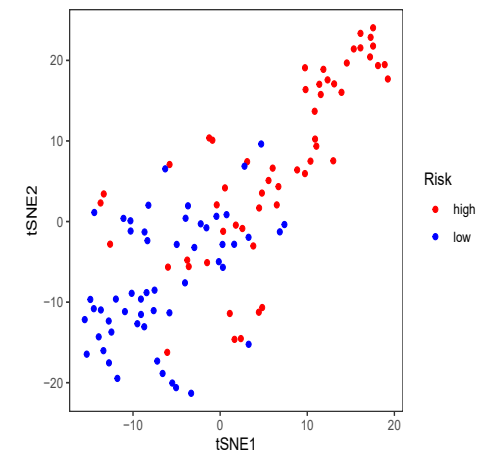

Supplement: Supplementary file 3 — Additional file 3: Figure S3. GSE26899 (A) and GSE26901 (B) survival curve. Survival status, time distribution, tSNE analysis, and PCA analysis between two different subgroups. [file 12967_2024_5109_MOESM3_ESM.pdf]

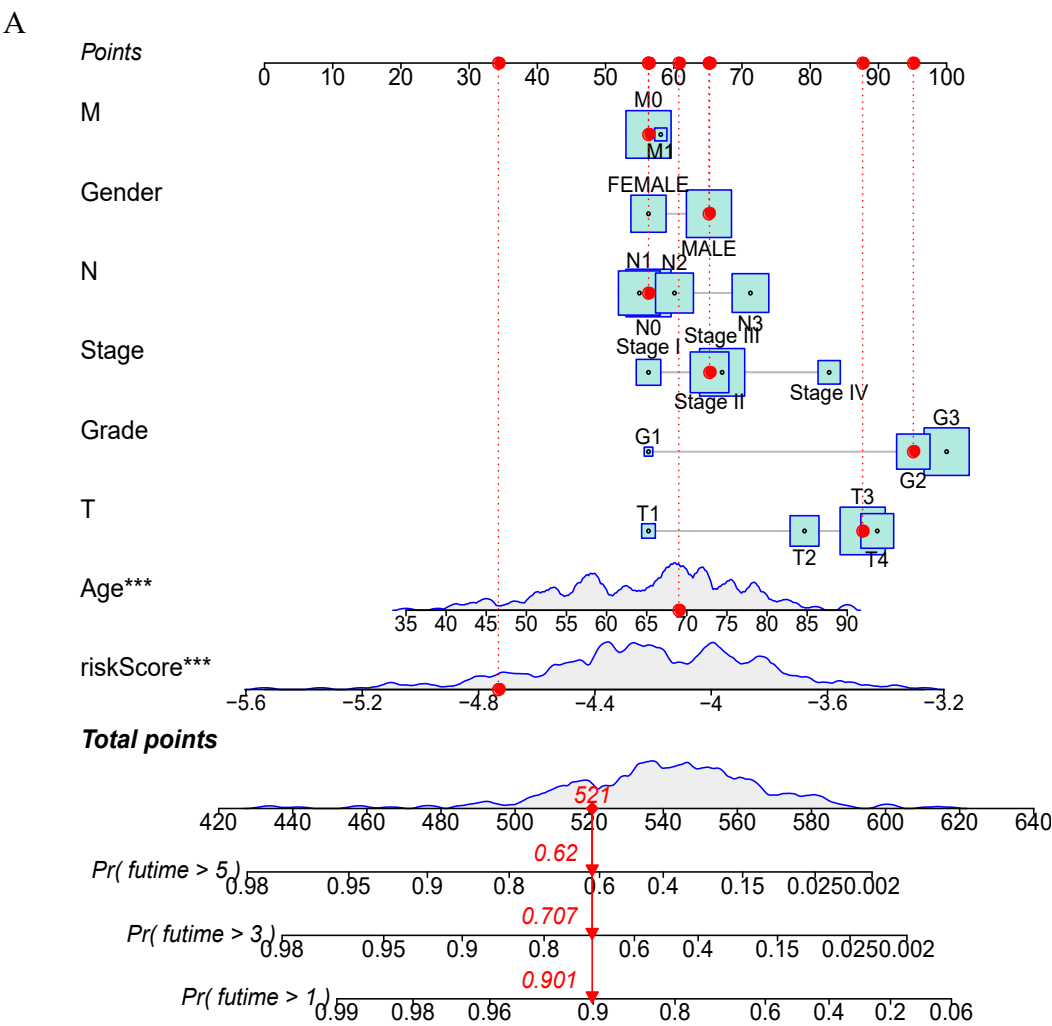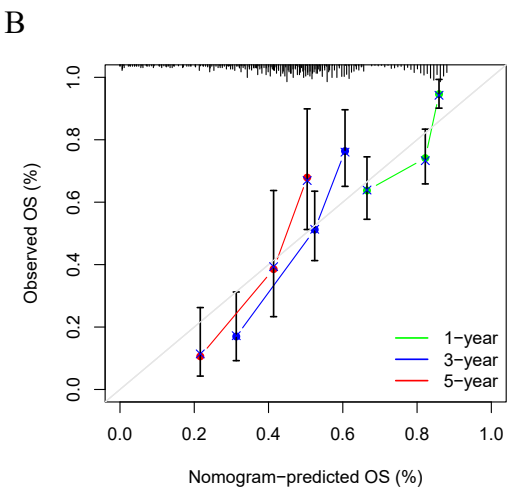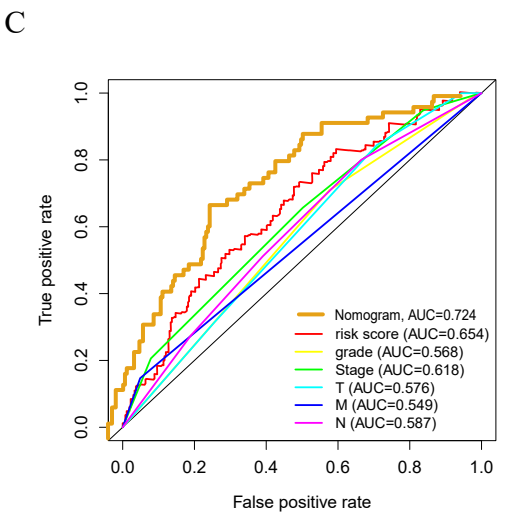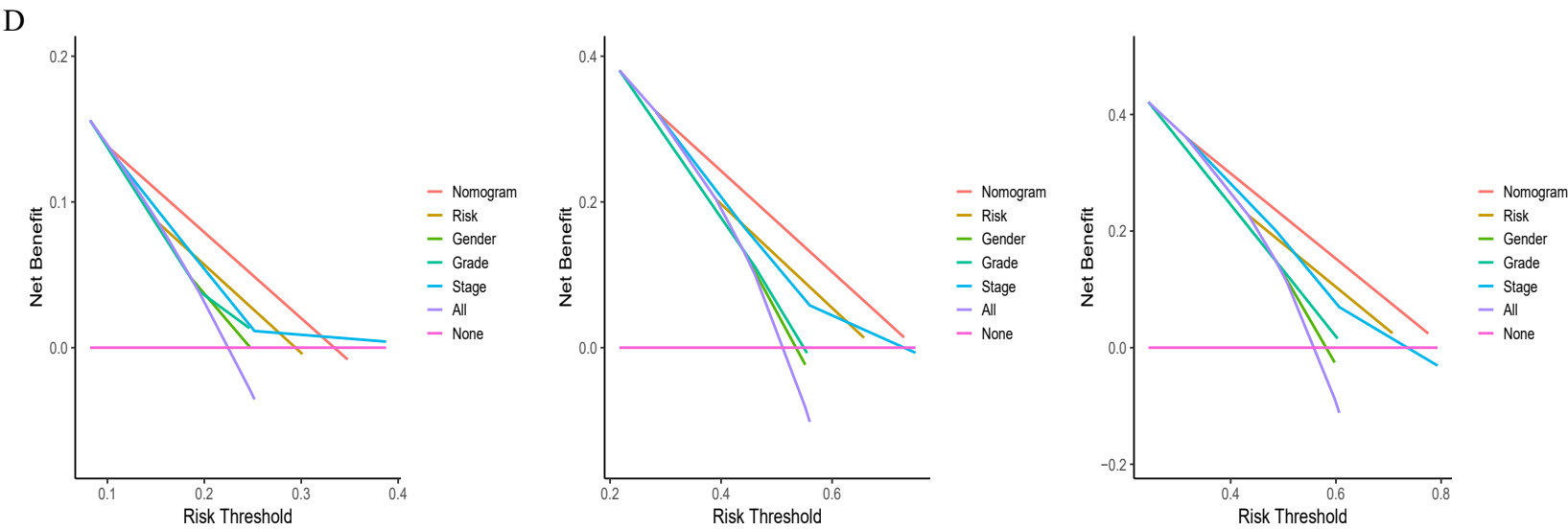

Supplement: Supplementary file 4 — Additional file 4: Figure S4. Building nomogram: A. Nomogram to predict 1-, 3-, and 5-year GC patient survival; B.Nomogram calibration curves for 1-, 3-, and 5-year OS; C. AUC analysis of each variable included in the nomogram model; D. DCA curves were compared over a period of 1 year, 3 years, and 5 years for patients with GC. [file 12967_2024_5109_MOESM4_ESM.pdf]

A

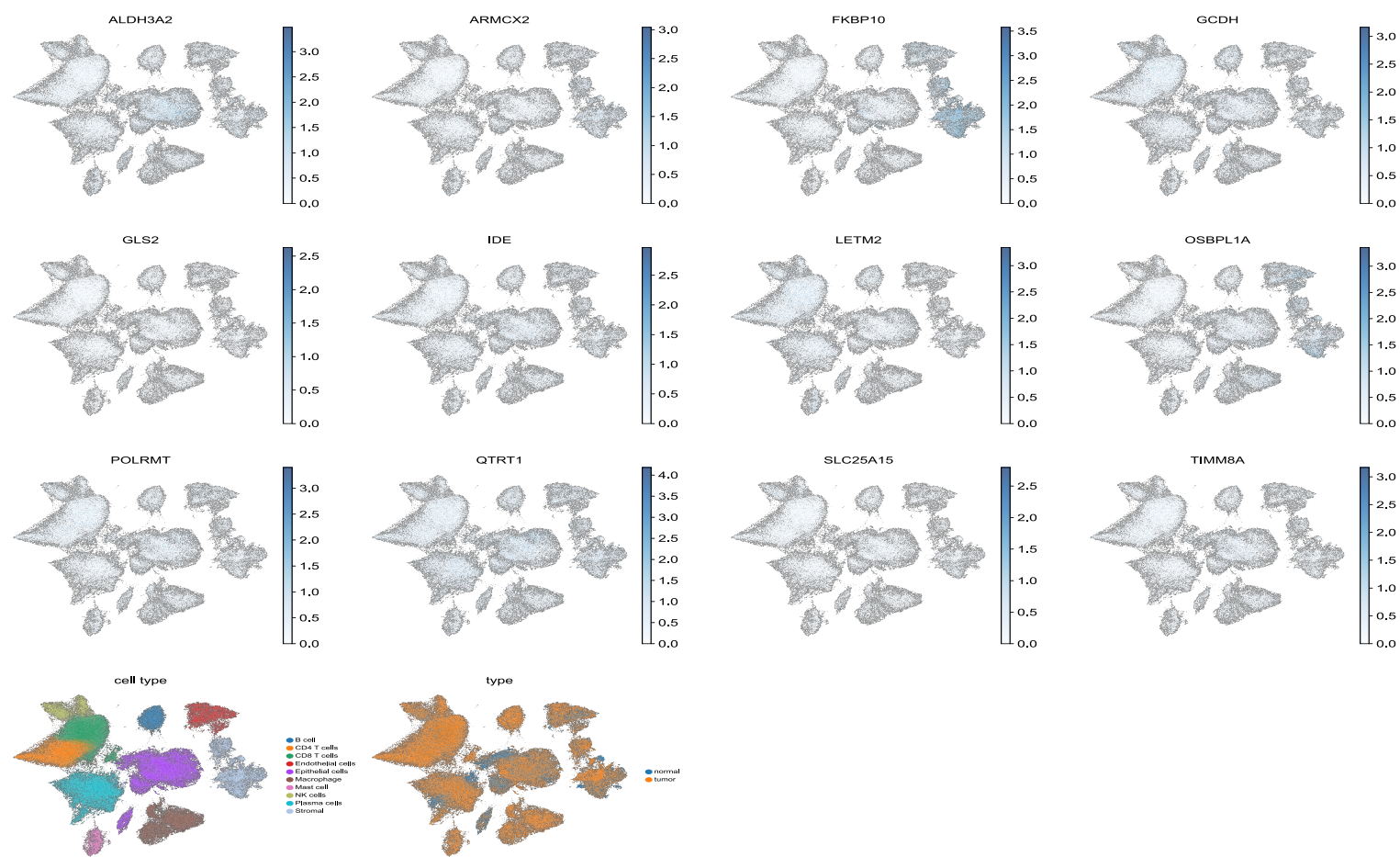

B

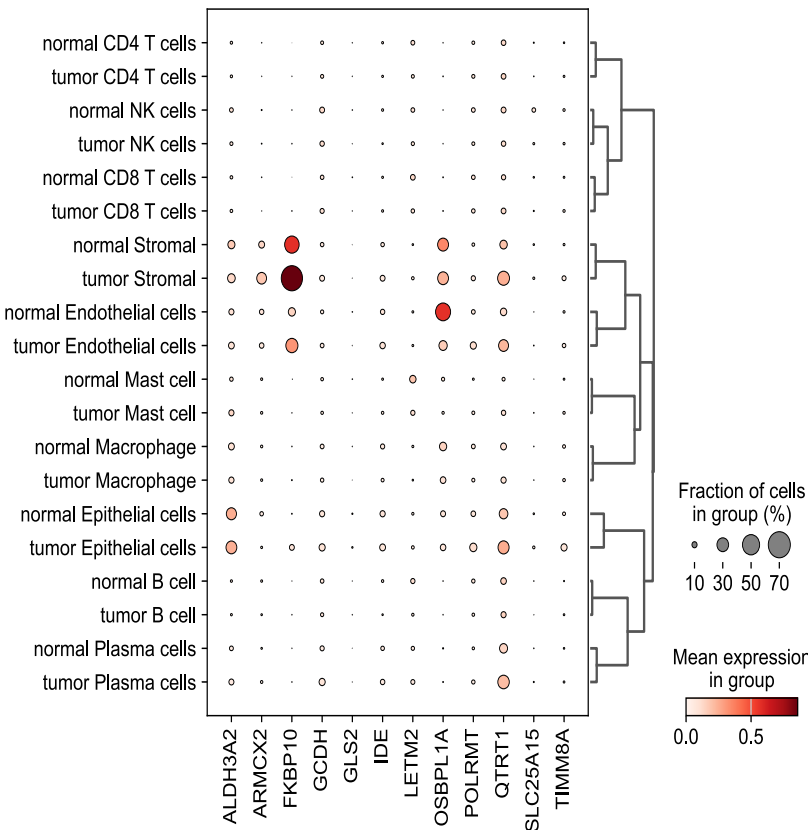

Supplement: Supplementary file 5 — Additional file 5: Figure S5. Uniform manifold approximation and projection (UMAP) plot of each MitoScore signature gene, cell types from gastric tumors cells from GEO: GSE183904. [file 12967_2024_5109_MOESM5_ESM.pdf]

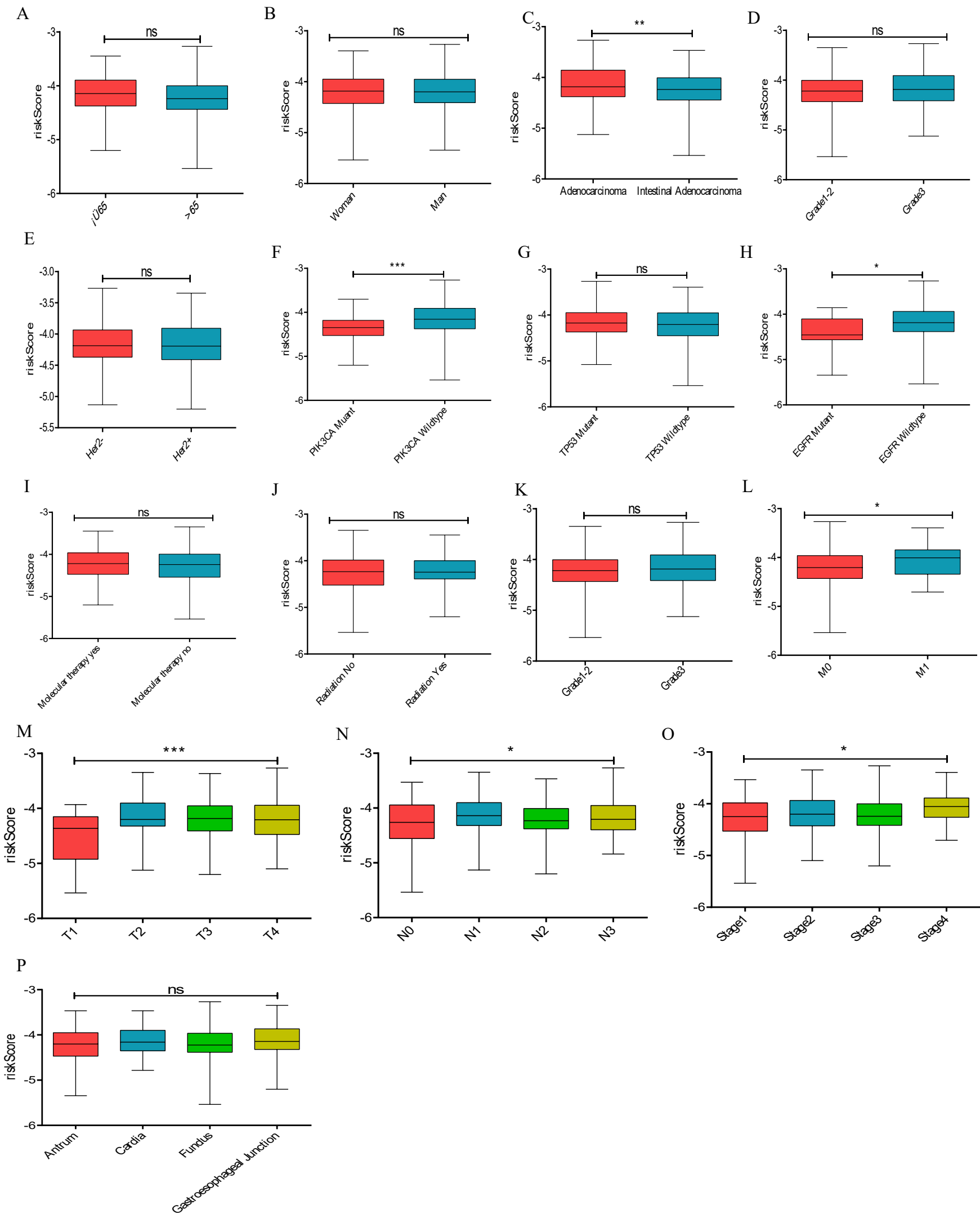

Supplement: Supplementary file 6 — Additional file 6: Figure S6. The expression of MitoScore signature in different clinical subgroups. [file 12967_2024_5109_MOESM6_ESM.pdf]

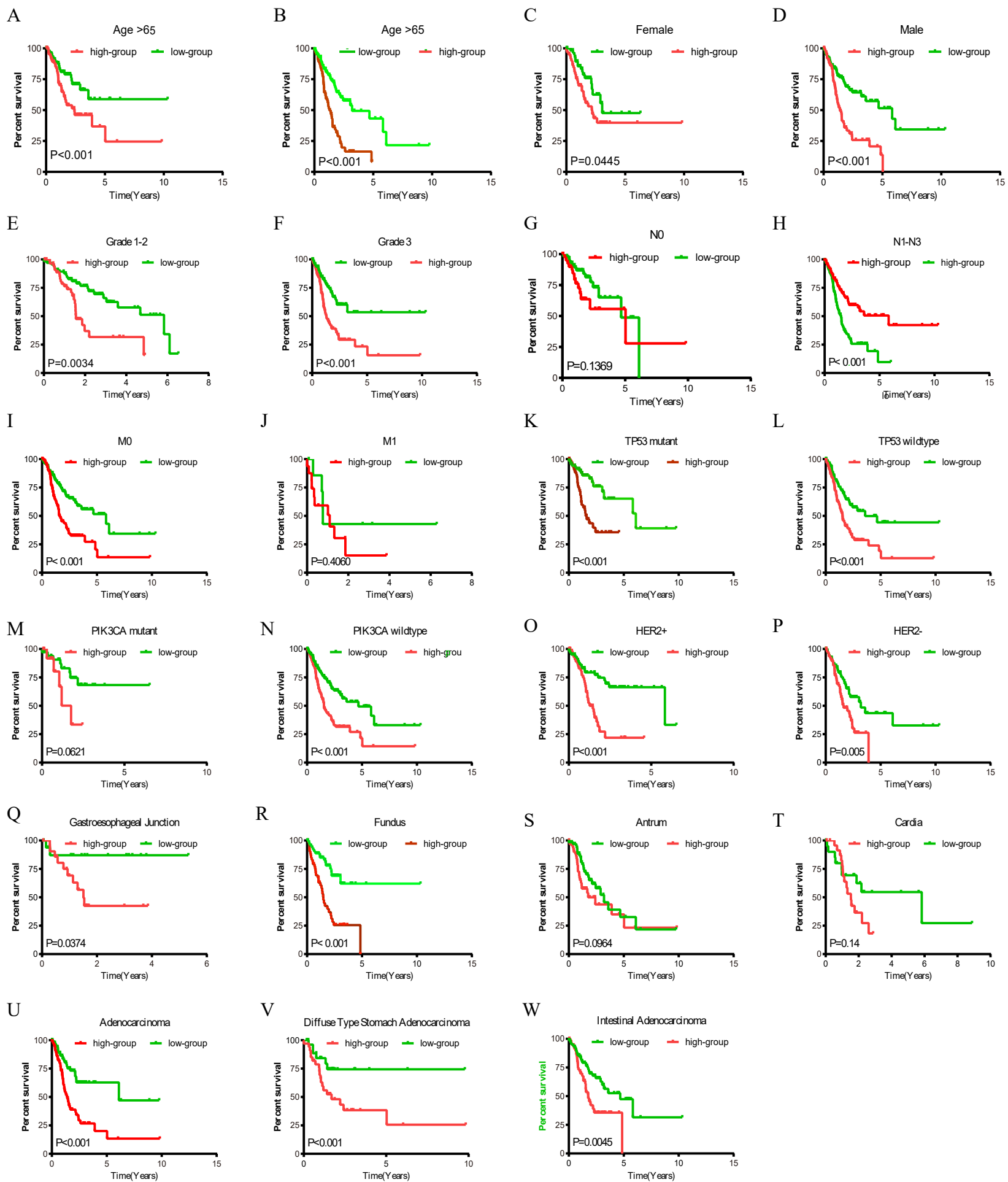

Supplement: Supplementary file 7 — Additional file 7: Figure S7. Kaplan-Meier survival analyses of MitoScore signature in different strata of clinical characteristics. [file 12967_2024_5109_MOESM7_ESM.pdf]

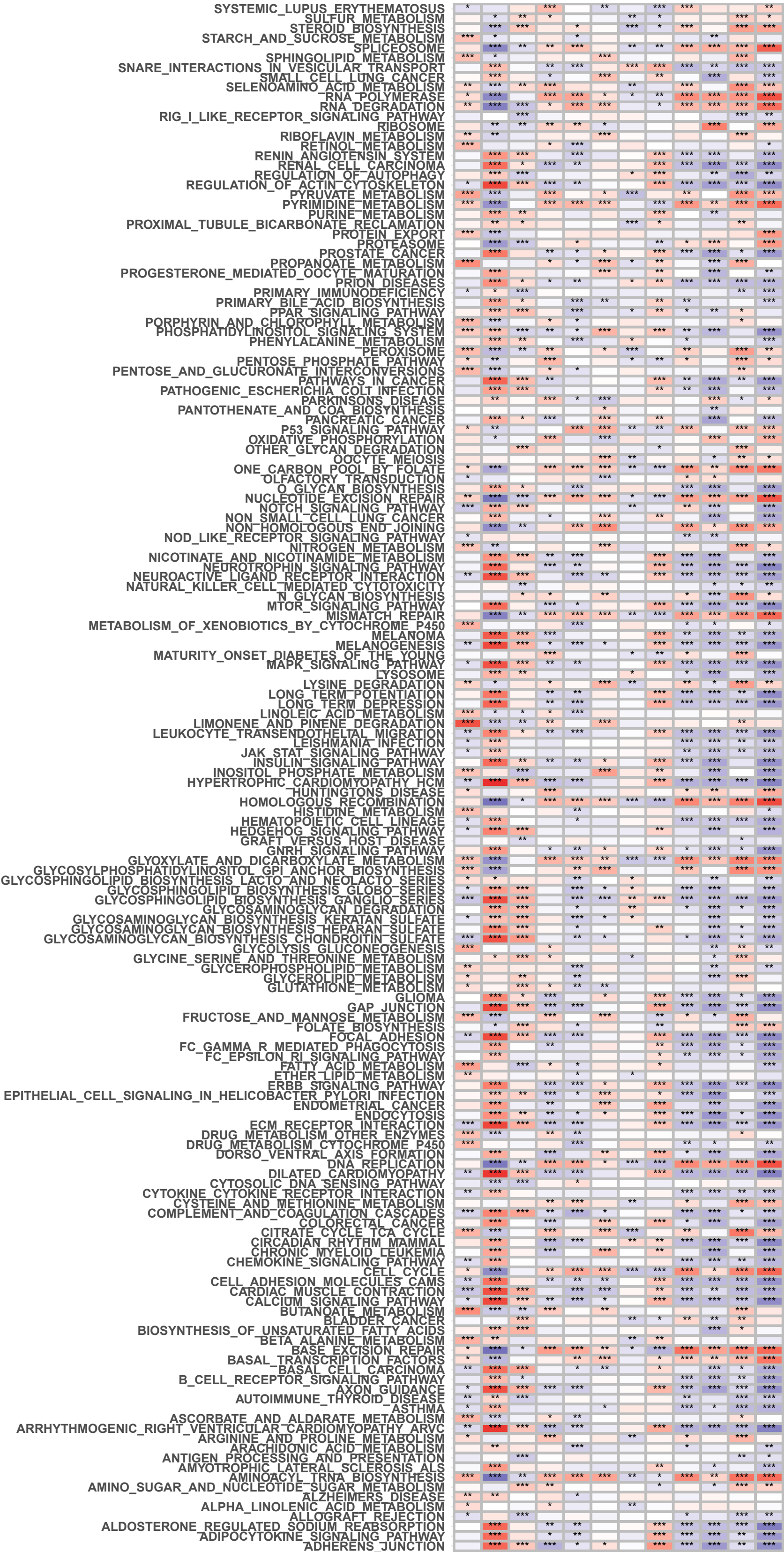

\*\*\* p<0.001  
\*\* p<0.01  
\* p<0.05

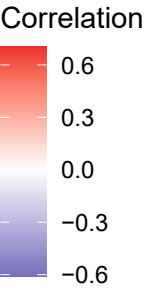

Supplement: Supplementary file 9 — Additional file 9: Figure S9. The heatmap of KEGG pathway activity of each MitoScore signature genes. [file 12967_2024_5109_MOESM9_ESM.pdf]

A

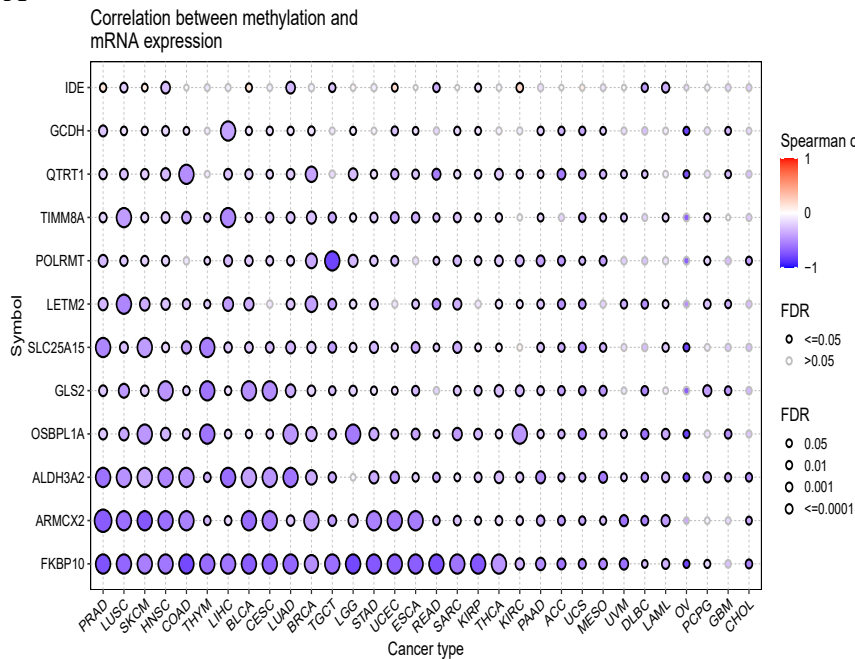

B

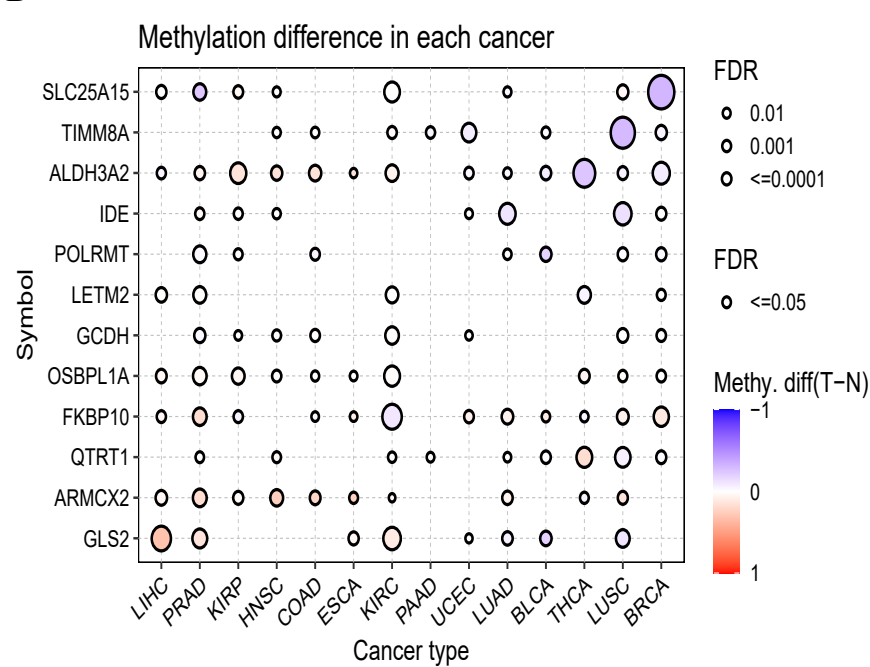

C

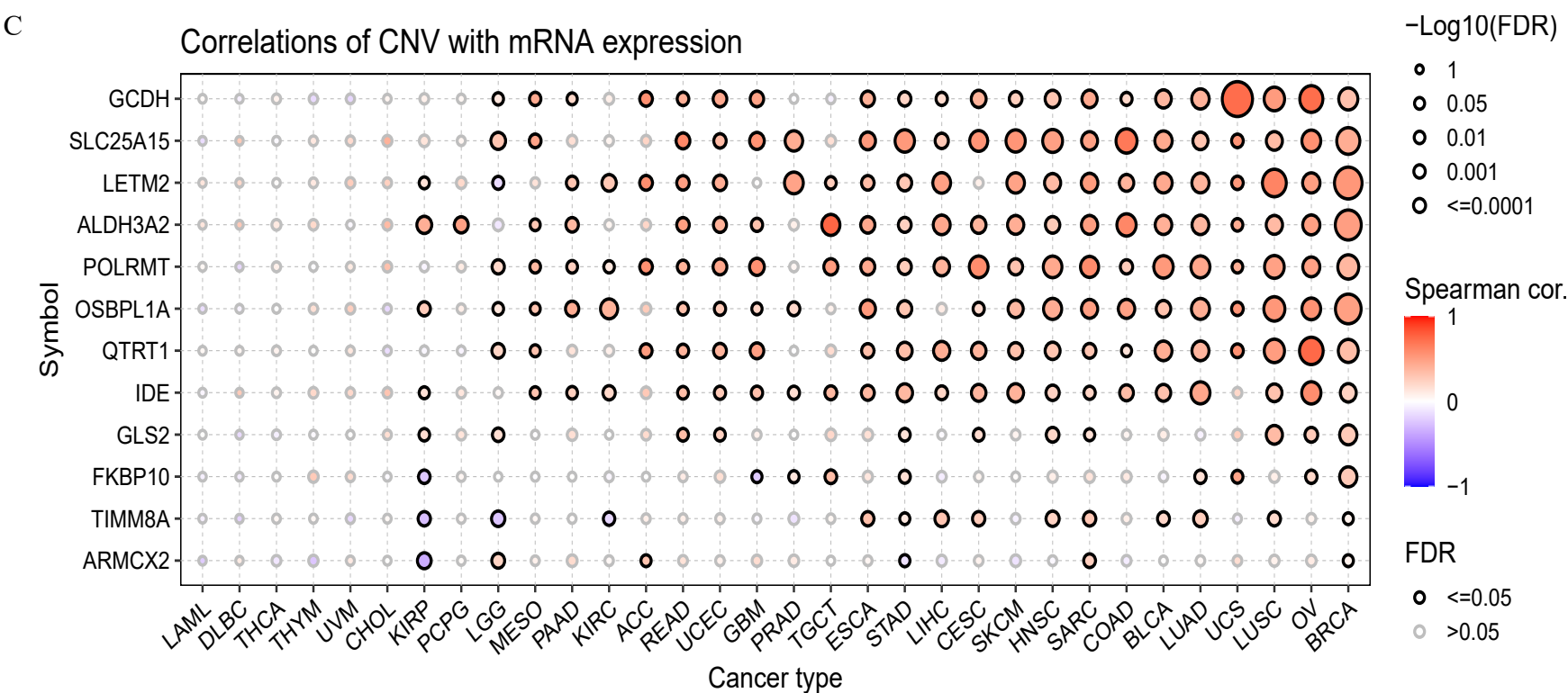

D

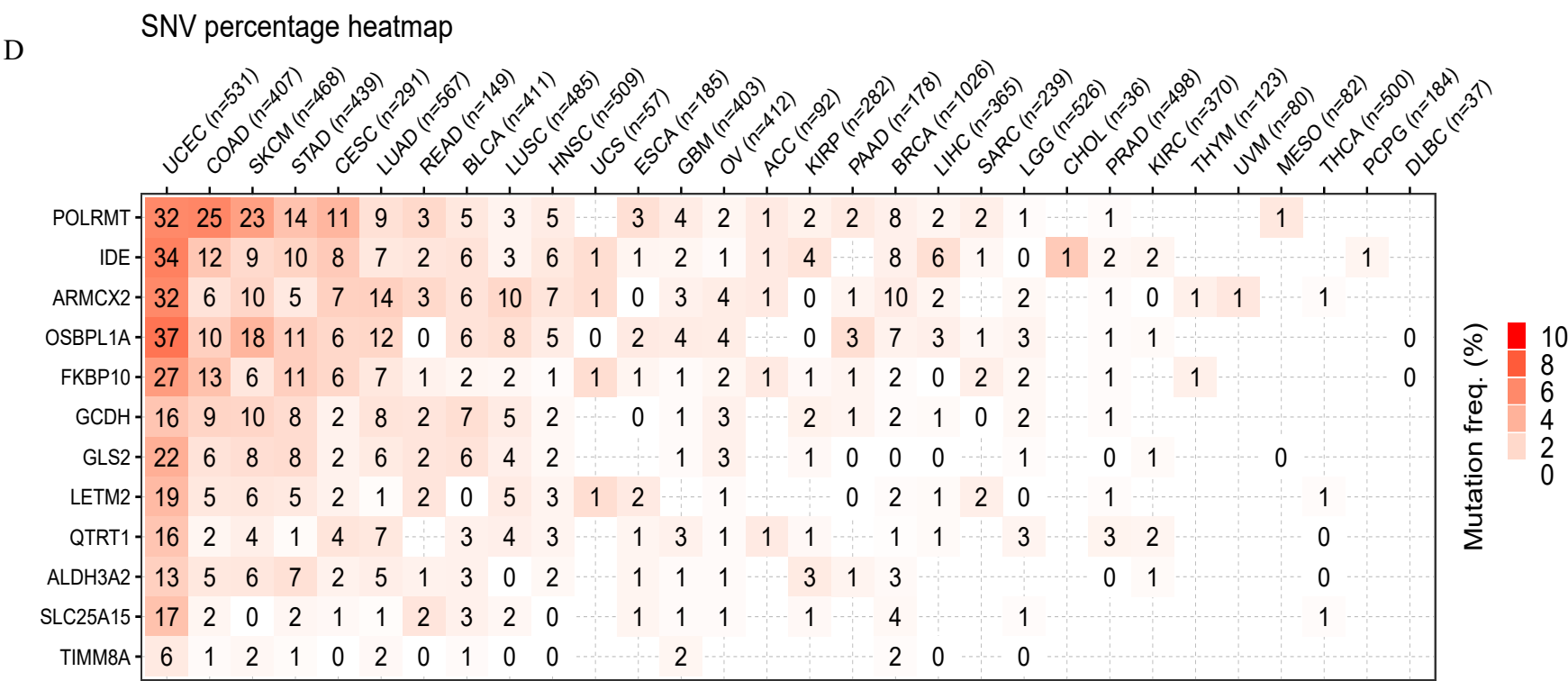

E

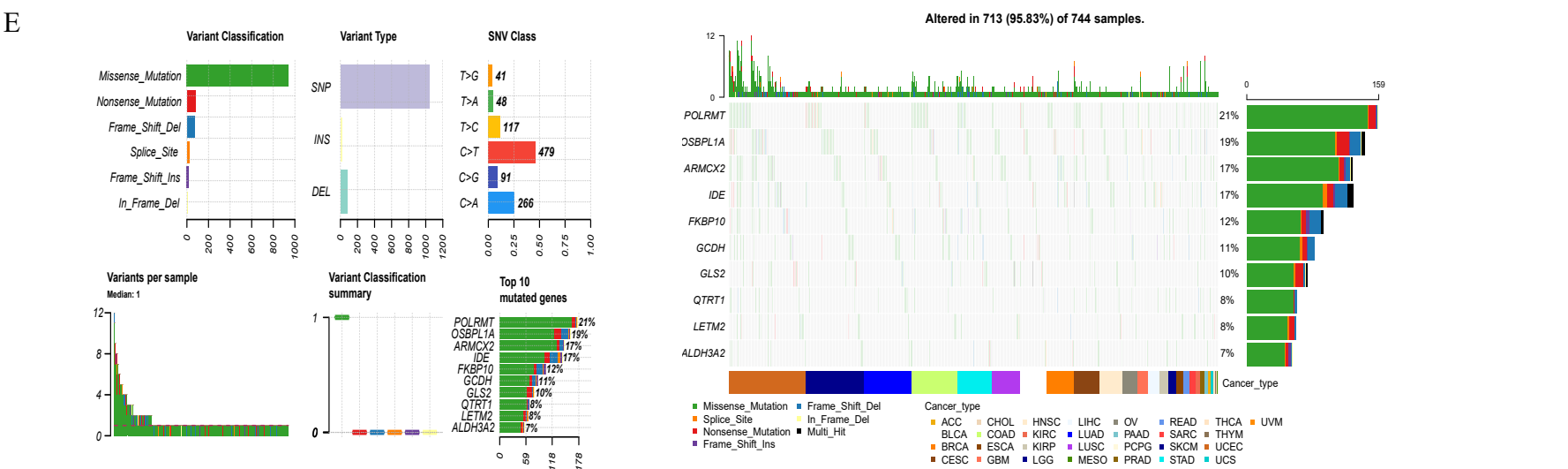

Supplement: Supplementary file 10 — Additional file 10: Figure S10. The landscape of MitoScore signature genes in pan-cancer analysis. A. Correlations between methylation and mRNA expression of inputted genes in the pan cancers; B. the methylation difference between tumor and normal samples in the pan cancers; C. correlations between CNV and mRNA expression in the pan cancers; D. copilot of the signature gene mutation frequency in pan cancers; E. copilot of the single-nucleotide variant in pan cancers. [file 12967_2024_5109_MOESM10_ESM.pdf]

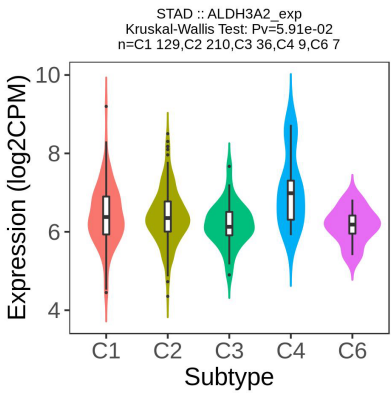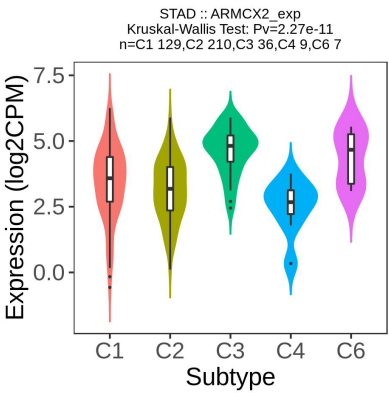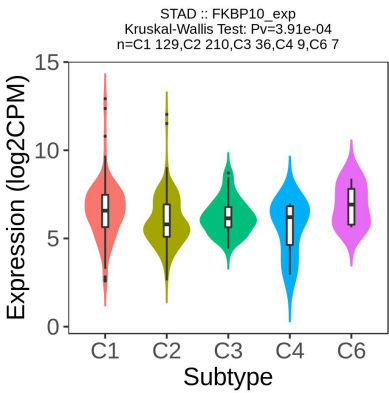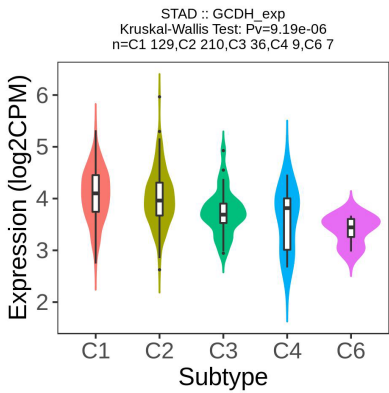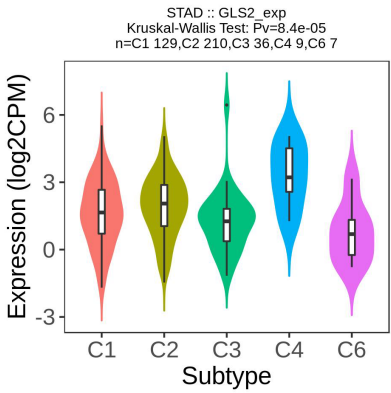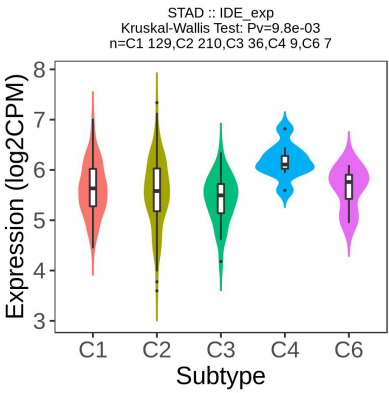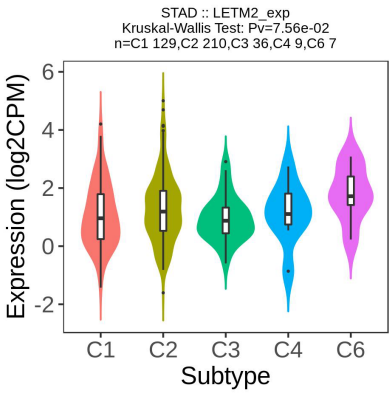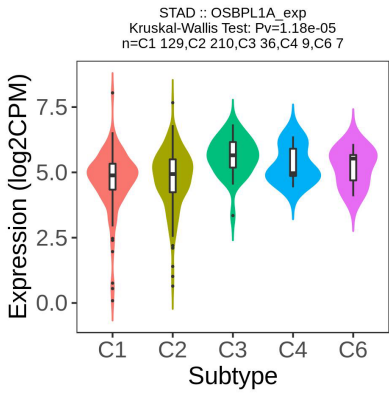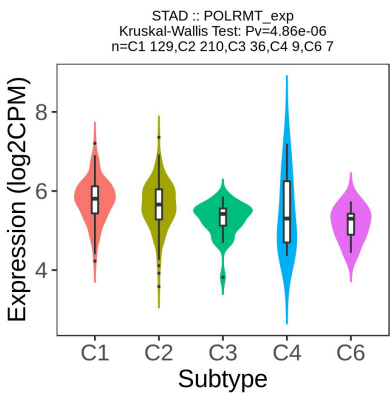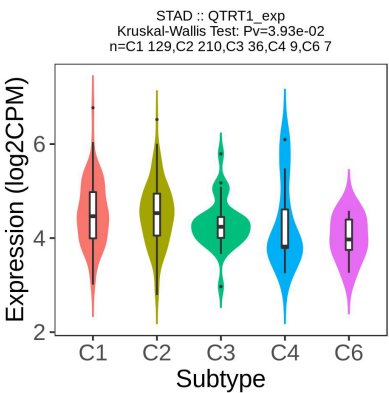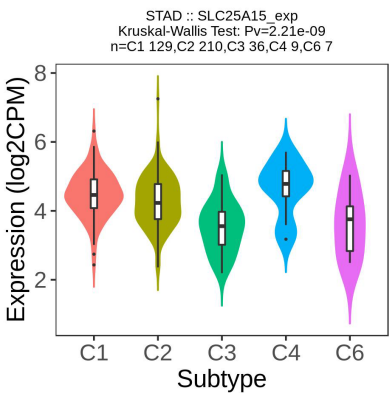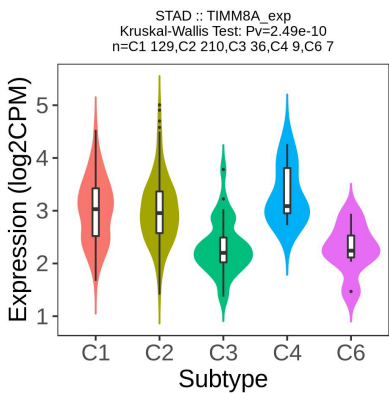

Supplement: Supplementary file 11 — Additional file 11: Figure S11. Box plot portrays the dissimilarities in the cancer immunity subgroup between MitoScore signature genes. [file 12967_2024_5109_MOESM11_ESM.pdf]

A

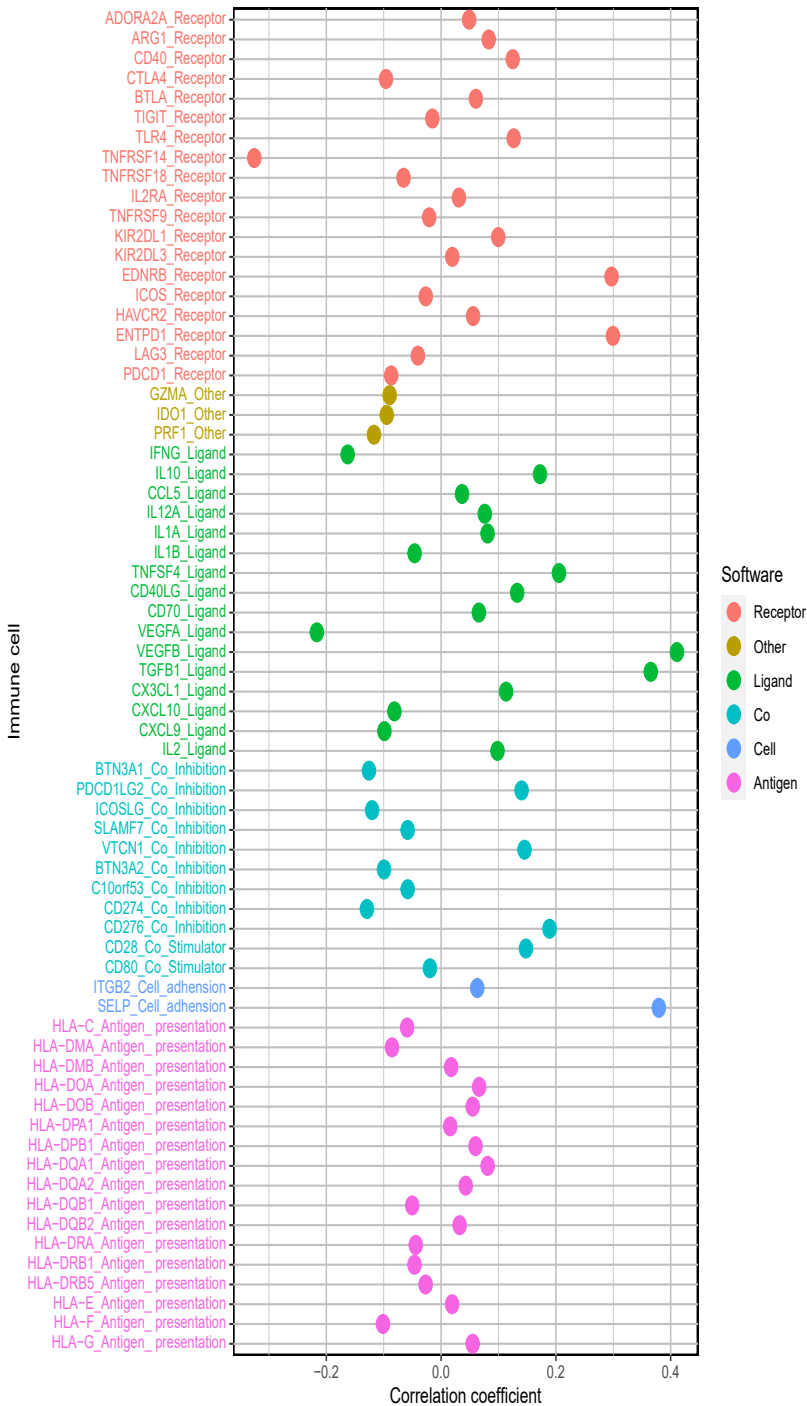

B

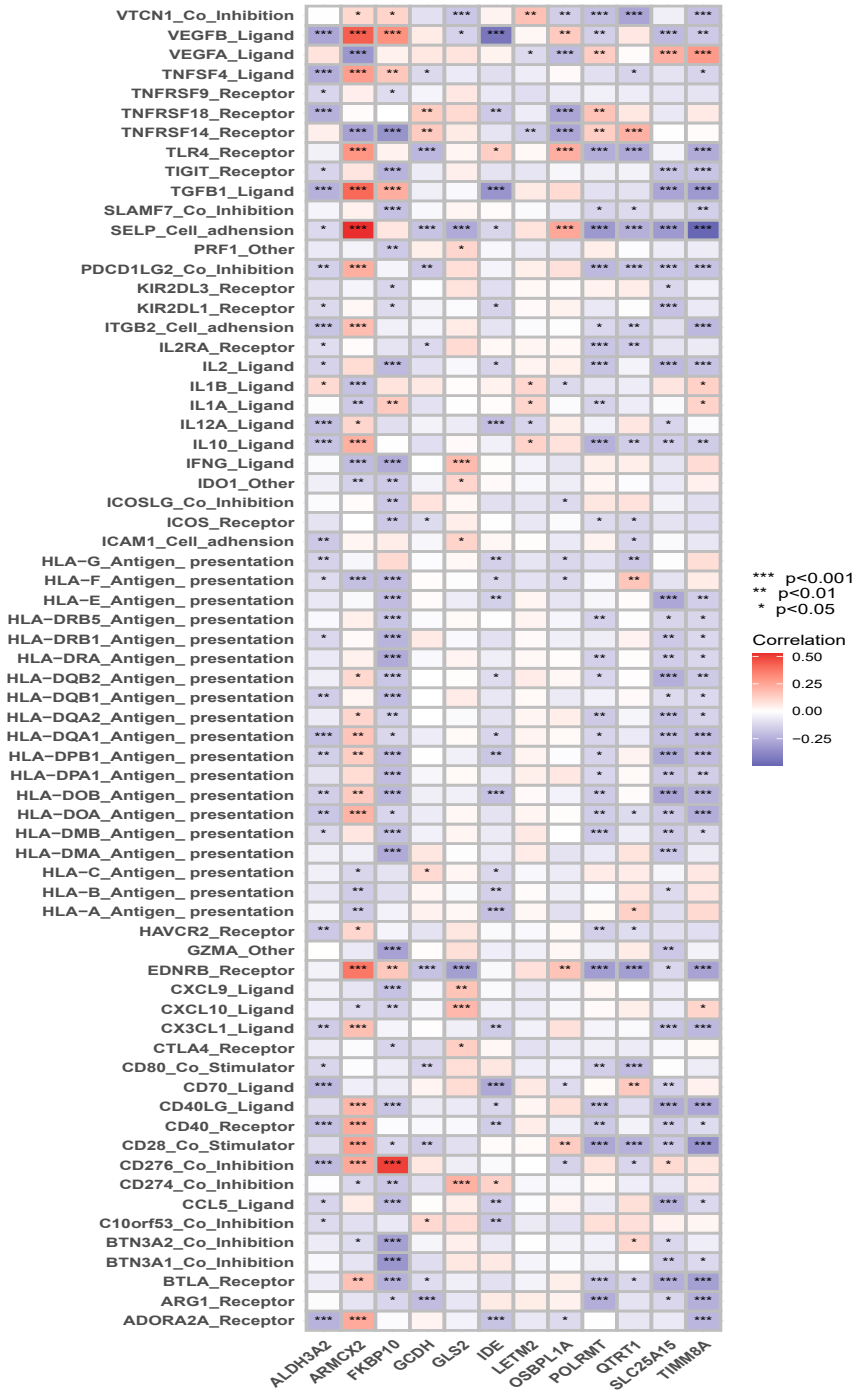

Supplement: Supplementary file 12 — Additional file 12: Figure S12. Correlation between the MitoScore and immune infiltration molecular. A. The correlation of immune molecular and MitoScore; B. Correlation analysis to estimate the presence of infiltrating immune molecular and MitoScore signature genes. [file 12967_2024_5109_MOESM12_ESM.pdf]

A

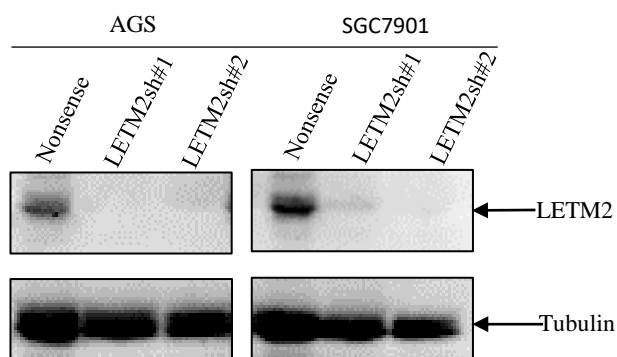

B

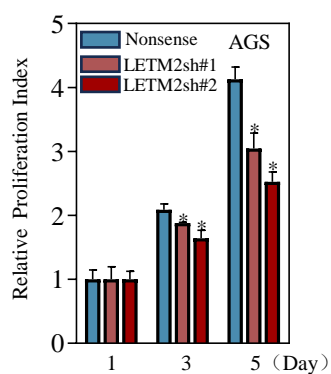

C

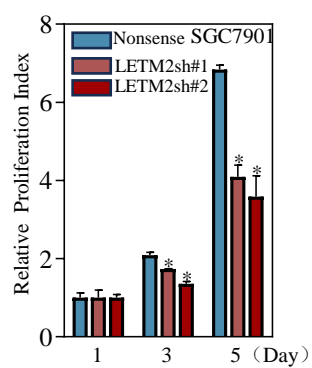

D

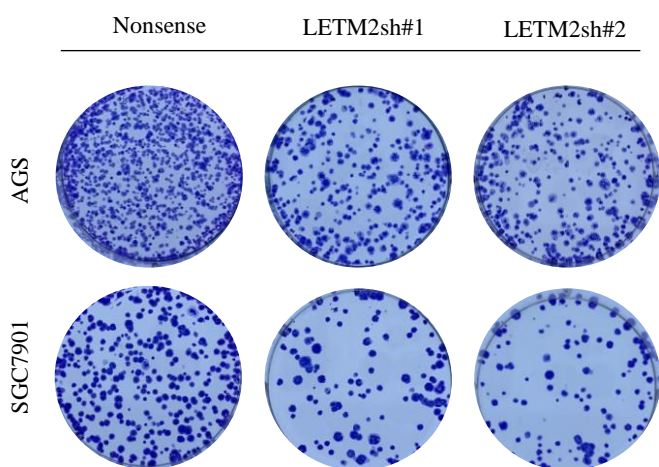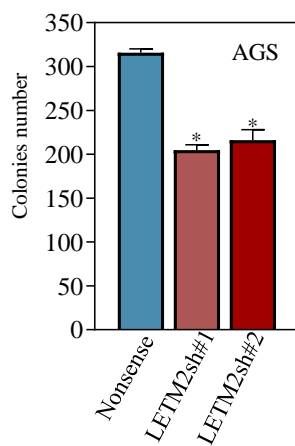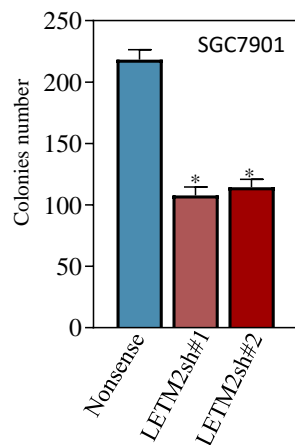

E

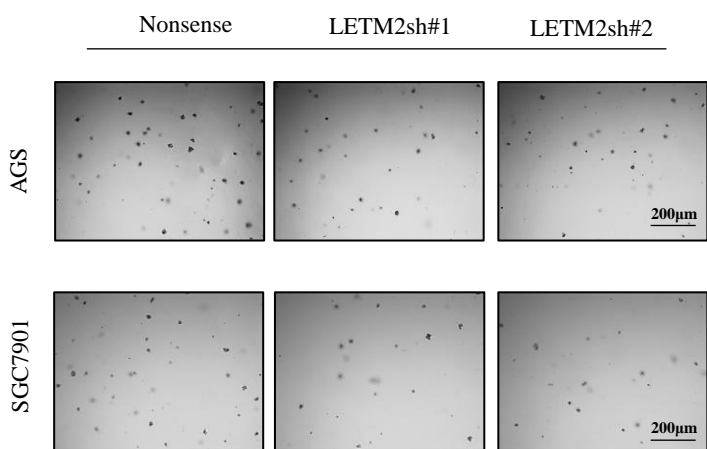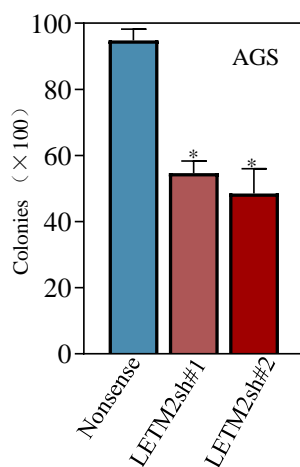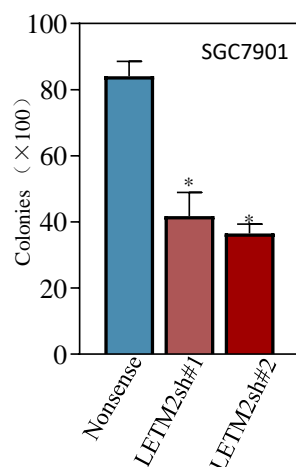

Supplement: Supplementary file 13 — Additional file 13: Figure S13. Knockdown of LETM2 inhibits the proliferative capacity of gastric cancer cells in vitro. (A) Knockdown LETM2 cell lines were identified. (B) ATP proliferation rate assay to detect the effect on AGS cell proliferation after knocking down LETM2. (B) ATP proliferation rate assay to detect the effect on AGS cell proliferation after knockdown of SGC7901. (D) Plate cloning assay to detect the effect on the proliferative ability of AGS and SGC7901 cells after knocking down LETM2. (E) soft agar assay to detect the effect on the anchorage-independent growth ability of AGS, SGC7901 cells after overexpression of LETM2. [file 12967_2024_5109_MOESM13_ESM.pdf]
