# Supplementary material for: Integrated multi-omics analysis and machine learning developed a prognostic model based on mitochondrial function in a large multicenter cohort for Gastric Cancer
Source: J Transl Med. 2024 Apr 23;22:381. doi: 10.1186/s12967-024-05109-7 (PMC11040813; doi:10.1186/s12967-024-05109-7)
Supplement: Supplementary file 8 — Additional file 8: Figure S8. KEGG pathway activity between high- and low-MitoScore subgroups based on ssGSEA algorithm. [file 12967_2024_5109_MOESM8_ESM.pdf]

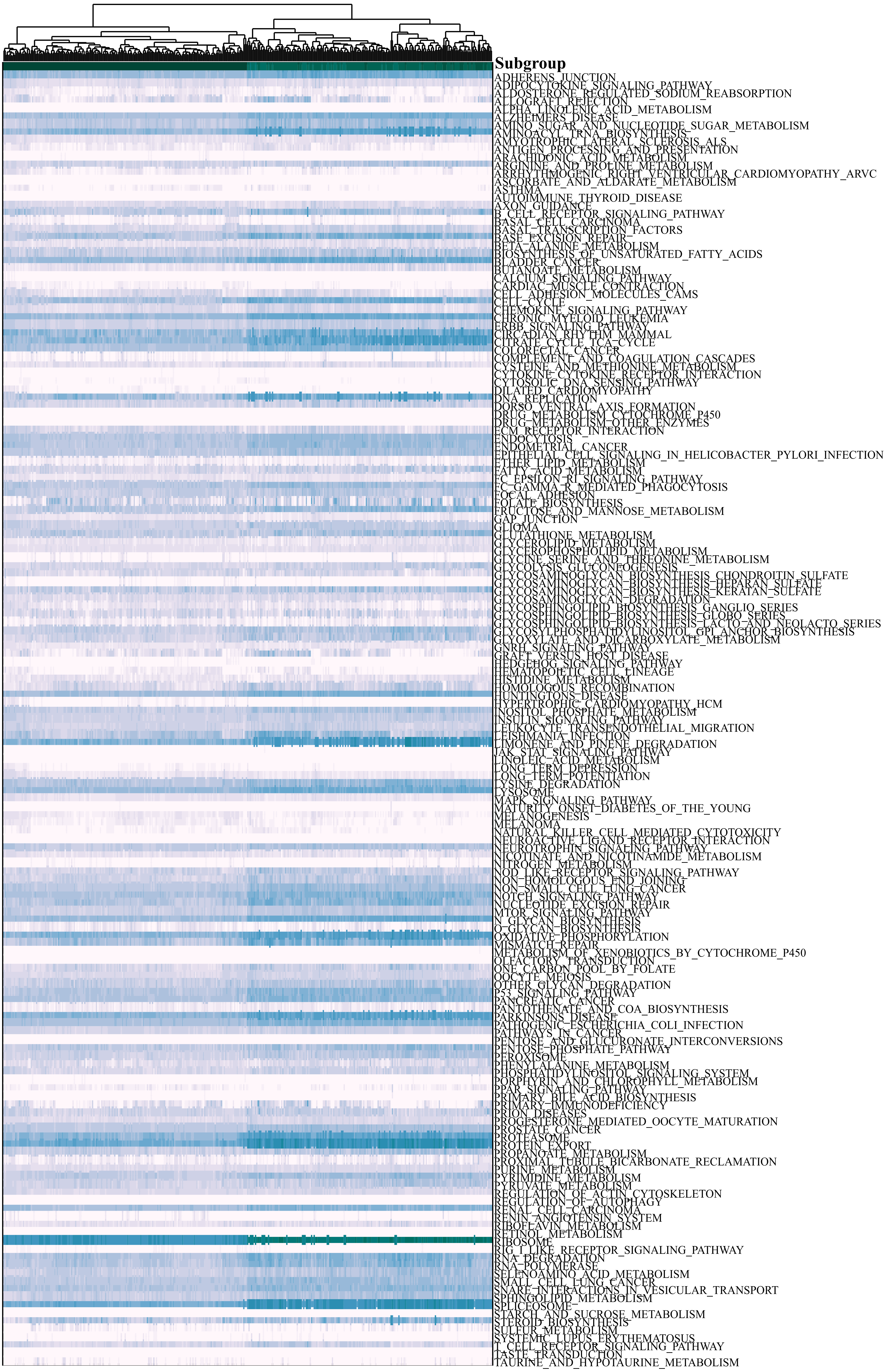
Subgroup
ADHERENS_JUNCTION
ADIPOCYTOKINE_SIGNALING_PATHWAY
ALDOSTERONE_REGULATED_SODIUM_REABSORPTION
ALLOGRAFT_REJECTION
ALPHA_LINOLENIC_ACID_METABOLISM
ALZHEIMERS_DISEASE
AMINO_SUGAR_AND_NUCLEOTIDE_SUGAR_METABOLISM
AMINOACYL_TRNA_BIOSYNTHESIS
AMYOTROPHIC_LATERAL_SCLEROSIS_ALS
ANTIGEN_PROCESSING_AND_PRESENTATION
ARACHIDONIC_ACID_METABOLISM
ARGININE_AND_PROLINE_METABOLISM
ARRHYTHMOGENIC_RIGHT_VENTRICULAR_CARDIOMYOPATHY_ARVC
ASCORBATE_AND_ALDARATE_METABOLISM
ASTHMA
AUTOIMMUNE_THYROID_DISEASE
AXON_GUIDANCE
B_CELL_RECEPTOR_SIGNALING_PATHWAY
BASAL_CELL_CARCINOMA
BASAL_TRANSCRIPTION_FACTORS
BASE_EXCISION_REPAIR
BETA_ALANINE_METABOLISM
BIOSYNTHESIS_OF_UNSATURATED_FATTY_ACIDS
BLADDER_CANCER
BUTANOATE_METABOLISM
CALCIUM_SIGNALING_PATHWAY
CARDIAC_MUSCLE_CONTRACTION
CELL_ADHESION_MOLECULES_CAMS
CELL_CYCLE
CHEMOKINE_SIGNALING_PATHWAY
CHRONIC_MYELOID_LEUKEMIA
ERBB_SIGNALING_PATHWAY
CIRCADIAN_RHYTHM_MAMMAL
CITRATE_CYCLE_TCA_CYCLE
COLORECTAL_CANCER
COMPLEMENT_AND_COAGULATION_CASCADES
CYSTEINE_AND_METHIONINE_METABOLISM
CYTOKINE_CYTOKINE_RECEPTOR_INTERACTION
CYTOSOLIC_DNA_SENSING_PATHWAY
DILATED_CARDIOMYOPATHY
DNA_REPLICATION
DORSO_VENTRAL_AXIS_FORMATION
DRUG_METABOLISM_CYTOCHROME_P450
DRUG_METABOLISM_OTHER_ENZYMES
ECM_RECEPTOR_INTERACTION
ENDOCYTOSIS
ENDOMETRIAL_CANCER
EPITHELIAL_CELL_SIGNALING_IN_HELICOBACTER_PYLORI_INFECTION
ETHER_LIPID_METABOLISM
FATTY_ACID_METABOLISM
FC_EPSILON_RI_SIGNALING_PATHWAY
FC_GAMMA_R_MEDIATED_PHAGOCYTOSIS
FOCAL_ADHESION
FOLATE_BIOSYNTHESIS
FRUCTOSE_AND_MANNOSE_METABOLISM
GAP_JUNCTION
GLIOMA
GLUTATHIONE_METABOLISM
GLYCEROLIPID_METABOLISM
GLYCEROPHOSPHOLIPID_METABOLISM
GLYCINE_SERINE_AND_THREONINE_METABOLISM
GLYCOLYSIS_GLUCONEOGENESIS
GLYCOSAMINOGLYCAN_BIOSYNTHESIS_CHONDROITIN_SULFATE
GLYCOSAMINOGLYCAN_BIOSYNTHESIS_HEPARAN_SULFATE
GLYCOSAMINOGLYCAN_BIOSYNTHESIS_KERATAN_SULFATE
GLYCOSAMINOGLYCAN_DEGRADATION
GLYCOSPHINGOLIPID_BIOSYNTHESIS_GANGLIO_SERIES
GLYCOSPHINGOLIPID_BIOSYNTHESIS_GLOBO_SERIES
GLYCOSPHINGOLIPID_BIOSYNTHESIS_LACTO_AND_NEOLACTO_SERIES
GLYCOSYLPHOSPHATIDYLINOSITOL_GPI_ANCHOR_BIOSYNTHESIS
GLYOXYLATE_AND_DICARBOXYLATE_METABOLISM
GNRH_SIGNALING_PATHWAY
GRAFT_VERSUS_HOST_DISEASE
HEDGEHOG_SIGNALING_PATHWAY
HEMATOPOIETIC_CELL_LINEAGE
HISTIDINE_METABOLISM
HOMOLOGOUS_RECOMBINATION
HUNTINGTONS_DISEASE
HYPERTROPHIC_CARDIOMYOPATHY_HCM
INOSITOL_PHOSPHATE_METABOLISM
INSULIN_SIGNALING_PATHWAY
LEUKOCYTE_TRANSENDOTHELIAL_MIGRATION
LEISHMANIA_INFECTION
LIMONENE_AND_PINENE_DEGRADATION
JAK_STAT_SIGNALING_PATHWAY
LINOLEIC_ACID_METABOLISM
LONG_TERM_DEPRESSION
LONG_TERM_POTENTIATION
LYSINE_DEGRADATION
LYSOSOME
MAPK_SIGNALING_PATHWAY
MATURITY_ONSET_DIABETES_OF_THE_YOUNG
MELANOGENESIS
MELANOMA
NATURAL_KILLER_CELL_MEDIATED_CYTOTOXICITY
NEUROACTIVE_LIGAND_RECEPTOR_INTERACTION
NEUROTROPHIN_SIGNALING_PATHWAY
NICOTINATE_AND_NICOTINAMIDE_METABOLISM
NITROGEN_METABOLISM
NOD_LIKE_RECEPTOR_SIGNALING_PATHWAY
NON_HOMOLOGOUS_END_JOINING
NON_SMALL_CELL_LUNG_CANCER
NOTCH_SIGNALING_PATHWAY
NUCLEOTIDE_EXCISION_REPAIR
MTOR_SIGNALING_PATHWAY
N_GLYCAN_BIOSYNTHESIS
O_GLYCAN_BIOSYNTHESIS
OXIDATIVE_PHOSPHORYLATION
MISMATCH_REPAIR
METABOLISM_OF_XENOBIOTICS_BY_CYTOCHROME_P450
OLFACTORY_TRANSDUCTION
ONE_CARBON_POOL_BY_FOLATE
OOCYTE_MEIOSIS
OTHER_GLYCAN_DEGRADATION
P53_SIGNALING_PATHWAY
PANCREATIC_CANCER
PANTOTHENATE_AND_COA_BIOSYNTHESIS
PARKINSONS_DISEASE
PATHOGENIC_ESCHERICHIA_COLI_INFECTION
PATHWAYS_IN_CANCER
PENTOSE_AND_GLUCURONATE_INTERCONVERSIONS
PENTOSE_PHOSPHATE_PATHWAY
PEROXISOME
PHENYLALANINE_METABOLISM
PHOSPHATIDYLINOSITOL_SIGNALING_SYSTEM
PORPHYRIN_AND_CHLOROPHYLL_METABOLISM
PPAR_SIGNALING_PATHWAY
PRIMARY_BILE_ACID_BIOSYNTHESIS
PRIMARY_IMMUNODEFICIENCY
PRION_DISEASES
PROGESTERONE_MEDIATED_OOCYTE_MATURATION
PROSTATE_CANCER
PROTEASOME
PROTEIN_EXPORT
PROPANOATE_METABOLISM
PROXIMAL_TUBULE_BICARBONATE_RECLAMATION
PURINE_METABOLISM
PYRIMIDINE_METABOLISM
PYRUVATE_METABOLISM
REGULATION_OF_ACTIN_CYTOSKELETON
REGULATION_OF_AUTOPHAGY
RENAL_CELL_CARCINOMA
RENIN_ANGIOTENSIN_SYSTEM
RIBOFLAVIN_METABOLISM
RETINOL_METABOLISM
RIBOSOME
RIG_I_LIKE_RECEPTOR_SIGNALING_PATHWAY
RNA_DEGRADATION
RNA_POLYMERASE
SELENOAMINO_ACID_METABOLISM
SMALL_CELL_LUNG_CANCER
SNARE_INTERACTIONS_IN_VESICULAR_TRANSPORT
SPHINGOLIPID_METABOLISM
SPLICEOSOME
STARCH_AND_SUCROSE_METABOLISM
STEROID_BIOSYNTHESIS
SULFUR_METABOLISM
SYSTEMIC_LUPUS_ERYTHEMATOSUS
T_CELL_RECEPTOR_SIGNALING_PATHWAY
TASTE_TRANSDUCTION
TAURINE_AND_HYPOTAURINE_METABOLISM
